# Laser-Induced Graphene Paper-Based Microfluidic Platform for Dual-Mode Electrochemical and Colorimetric Detection of Glucose

**DOI:** 10.1021/acsomega.6c09156

**Published:** 2026-10-24

**Authors:** Che-Liang Yeh, Syed Atif Ali, Wen-Ti Wu, Hsiung-Lin Tu

**Affiliations:** Institute of Chemistry, 71552 Academia Sinica, Nankang, Taipei 11529, Taiwan

## Abstract

Microfluidic paper-based analytical devices (μPADs) provide an attractive platform for diagnostics owing to their low cost, passive fluid transport, and ease of fabrication. However, integrating dual-sensing capabilities into a single paper device while maintaining a simple manufacturing process remains challenging. Here, we present a foldable dual-mode microfluidic paper-based analytical device (DM-μPAD) that integrates laser-induced graphene (LIG)-based electrochemical sensing with enzyme-mediated colorimetric detection. A straightforward laser-patterning strategy was used to fabricate LIG electrodes directly patterned onto the polyimide tape-covered cellulose paper via a one-step laser engraving process, forming a conductive graphene network that serves as an electrochemical sensing module. A screen-printing process was used to form hydrophobic perfluoropolyether barriers, which define capillary-driven microfluidic channels that guide sample transport between sensing regions. The folded architecture enhances fluid-electrode interaction and enables simultaneous electrochemical and colorimetric measurements within a single analytical platform without electrode transfer or complex assembly. This dual-readout technique provides cross-validation of results to enhance detection reliability. The proposed DM-μPAD demonstrated good selectivity against common nonspecific compounds, a wide linear detection range of 0.01 to 3.0 mM, and a low detection limit of 9.23 μM in the electrochemical mode, while displaying a dynamic detection range of 0.1 to 2.0 mM with a detection limit of 57.9 μM in the colorimetric mode. Furthermore, the platform maintained high reproducibility, mechanical flexibility, and environmental stability. This work highlights the potential of laser-induced graphene-based paper microfluidics as a versatile platform for developing portable, cost-effective, and multiplexed biosensing devices.

## Graphical abstract

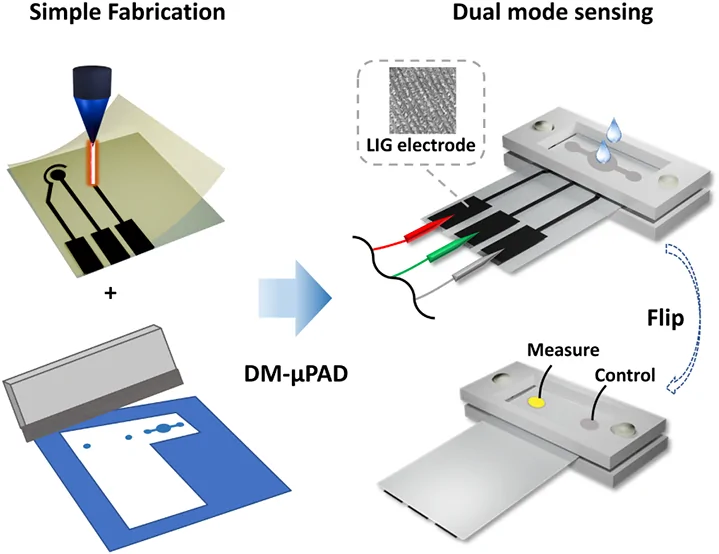

## 1 Introduction

According to reports from the World Health Organization (WHO), the global prevalence of diabetes has increased dramatically over the past few decades, largely driven by unhealthy dietary habits and physical inactivity.^1^ Diabetes not only increases the risk of severe complications, including cardiovascular and kidney diseases, but also imposes a substantial socioeconomic burden on individuals and healthcare systems.^2^ Effective glucose monitoring is therefore essential for disease management and the prevention of diabetes-related complications.^3^ Although continuous glucose monitoring technologies provide dynamic assessment of glucose levels, conventional blood glucose meters remain widely used and require repeated finger-pricking, causing discomfort, and increasing the risk of infection.^4^ Consequently, there is growing interest in developing noninvasive, portable sensing technologies capable of continuous glucose monitoring.^5,6^ To address these limitations, alternative noninvasive biofluids such as sweat,^7,8^ saliva,^9,10^ and urine^11,12^ have been explored for glucose monitoring. Among them, sweat has gained considerable attention due to its rich composition of biomarkers, including metabolites, electrolytes, and proteins that reflect physiological conditions.^13–15^ Notably, several studies have demonstrated a positive correlation between sweat glucose and blood glucose levels, indicating its potential for glucose monitoring.^16–18^ Sweat-based sensing offers key advantages, including noninvasive sampling, minimal discomfort, and the possibility of continuous monitoring through portable devices. However, translating sweat glucose sensing from the laboratory to practical applications remains challenging because glucose is present in sweat at relatively low concentrations, requiring sensing platforms with high sensitivity and reliable analytical performance.^19^ Another major limitation is the difficulty in reliable sweat collection, as sweat secretion often requires stimulation through physical activity or elevated temperatures, and conventional collection methods lack reproducibility.^20^ Furthermore, the wide physiological range of glucose concentrations in sweat (10–1000 μM) demands sensing platforms with high sensitivity and a wide linear response range.^2,3^ In recent years, three-dimensional (3D) μPADs have emerged as promising platforms for biosensing applications due to their low cost, flexibility, portability, and ease of fabrication.^21–23^ These devices utilize capillary-driven flow to enable reagent transport without external power, making them highly suitable for point-of-care testing (POCT) and portable sensing applications.^24^ By integrating multiple detection functionalities, such as electrochemical and colorimetric methods, paper-based microfluidics offer enhanced analytical performance and user-friendly operation.

Despite significant advances in μPAD technology, integrating multiple sensing modalities within a single platform while maintaining simple fabrication, reliable fluid transport, and scalable manufacturing remains challenging. Most reported paper-based devices rely on multiple fabrication steps, including electrode transfer, conductive ink printing, metal sputtering, or assembly of individual sensing components. These approaches increase fabrication complexity, reduce manufacturing reproducibility, and may compromise the inherent advantages of paper microfluidics. Furthermore, integrating electrochemical electrodes without disrupting capillary-driven flow remains a persistent challenge in paper-based devices. Conventional electrode materials, including noble metals (such as Au and Pt) and metal oxides (such as ZnO, TiO_2_, etc.), have been extensively employed in electrochemical sensing owing to their excellent electrical conductivity, electrocatalytic activity, chemical stability, and favorable surface chemistry. However, noble-metal electrodes are associated with high material and fabrication costs, limiting their applicability in low-cost and disposable sensing platforms. In contrast, metal oxides offer low-cost and high-surface-area architectures with tunable catalytic properties, but their relatively poor electrical conductivity, structural instability under certain conditions, and susceptibility to matrix-related interference can compromise electron-transfer efficiency, long-term stability, and reproducibility. These limitations have motivated the development of alternative electrode materials with improved electrical performance, cost-effectiveness, structural tunability, and compatibility with miniaturized and flexible sensing platforms.^25,26^ In contrast, carbon-based materials, including graphene and porous carbon, have attracted significant attention due to their excellent conductivity, large surface area, chemical stability, and biocompatibility.^27–29^ Among these, laser-induced graphene (LIG) has emerged as a versatile and scalable material, enabling the direct conversion of carbon-rich substrates with heat resistance such as polyimide into porous graphitic structures via laser engraving. This technique offers precise patterning, high conductivity, and a large density of active sites, making it suitable for electrochemical sensing applications.^30^ Recently, many studies haveexplored the direct conversion of insulating polymers (such as polyimide) into conductive materials using a CO_2_ laser, or even converting flame retardant treated paper into electrodes. However, these electrodes are often hydrophobic, which hinders capillary flow, and display limited contact with the fluid within pores.^31^ Although research indicates that the retardant can be washed away, the fragmentation of paper may cause small damage during the washing process, leading to changes in capillary flow. Therefore, developing a fabrication strategy that directly integrates conductive electrodes with paper microfluidics while preserving capillary flow remains desirable.

In this work, we propose a foldable 3D DM-μPAD fabricated through a simple laser-patterning and screen-printing strategy (Scheme 1). The 3D channel was designed to ensure that sample flow is not obstructed by the electrodes. By employing controlled laser engraving, a porous and conductive graphene network is directly patterned onto the polyimide tape-covered paper, enabling efficient electrochemical sensing with a linear detection range of 0.01 to 3.0 mM. Furthermore, the folded architecture incorporates dual-mode detection by combining electrochemical analysis with colorimetric sensing, providing both quantitative and visual readouts. This integrated approach offers simple fabrication, integrated fluid handling, and a low-cost, portable, and reliable solution for noninvasive glucose monitoring, with significant potential for portable and point-of-care applications.

**Scheme 1:**
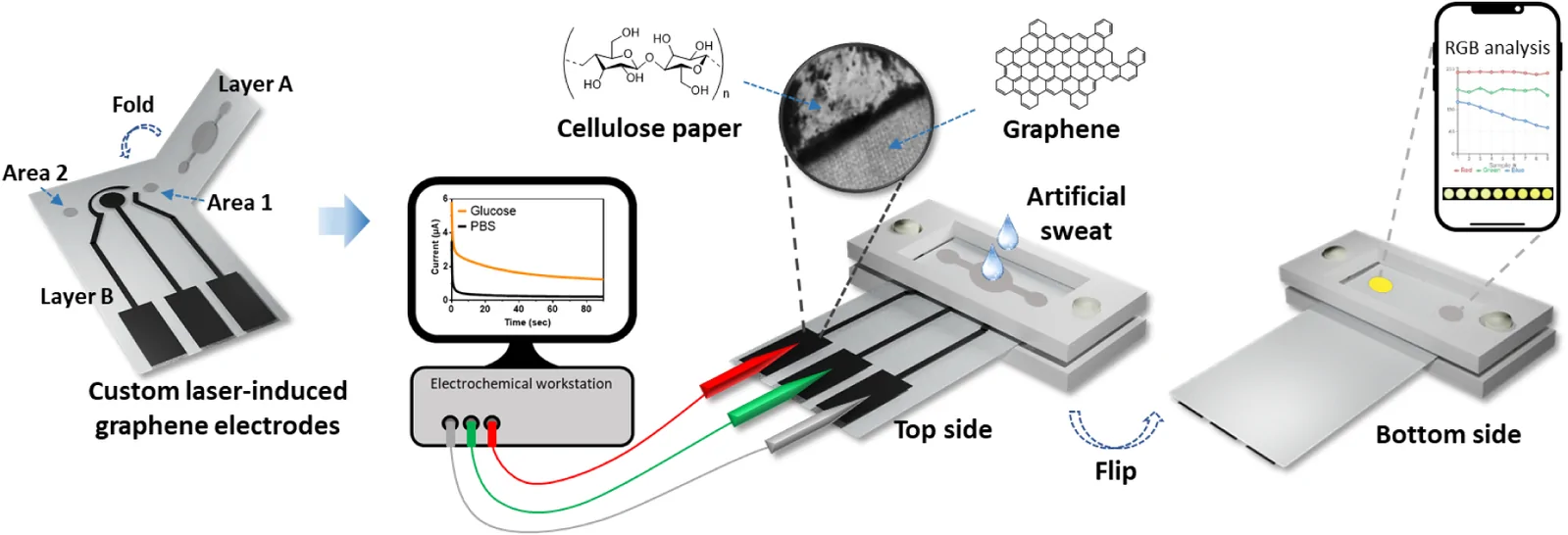
Schematic Illustration of the Developed DM-μPAD Device and Its Working Principle

## 2 Experimental Section

### 2.1 Reagents and Instruments

d-(+)-Glucose (Fisher Chemical, USA), glucose oxidase (GOx) (Sigma-Aldrich, USA), chitosan oligosaccharide lactate (Sigma-Aldrich, USA), horseradish peroxidase (HRP) (Sigma-Aldrich, USA), *o*-phenylenediamine (OPD) (Tokyo Chemical Industry, Japan), lactose (Sigma-Aldrich, USA), sucrose (Fisher Chemical, USA), urea (Fisher Chemical, USA), ascorbic acid (AA) (Fisher Chemical, USA), dopamine (DA) (AK Scientific, USA), perfluoropolyether-urethane dimethacrylate (Fluorolink MD700), 2-hydroxy-2-methylpropiophenone (Darocur 1173), polydimethylsiloxane (PDMS), crayon, paraffin wax, polyimide tape, potassium hexacyanoferrate­(III) (K_3_[Fe­(CN)_6_]), potassium hexacyanoferrate­(II) trihydrate (K_4_[Fe­(CN)_6_]·3H_2_O), potassium chloride, and phosphate-buffered saline (PBS) with pH 7.4. All chemicals were of analytical grade. Cellulose paper (ADVANTEC filter paper, grade 1) was used as the substrate for device fabrication. The electrode was designed using Autodesk Fusion (Autodesk, USA) and fabricated by CO_2_ laser system (GCC LaserPro C180, Taiwan) (wavelength: 10640 nm). All aqueous solutions were prepared using 0.1 M PBS solution and deionized (DI) water (resistivity ≥ 18.2 MΩ·cm). All electrochemical measurements, comprising chronoamperometry (CA) and cyclic voltammetry (CV), were carried out employing an electrochemical workstation (CHI4054C, CH Instruments, USA). Scanning electron microscopy (FE-SEM, ZEISS ULTRA PLUS, Germany) and Raman spectroscopy (Nanobase XperRAM-S, Korea) (wavelength: 532 nm) were used to characterize the electrode surface morphology. A contact angle analyzer (PSC-1000B, PENTAD, Taiwan) was used to characterize the wetting behavior of various materials.

### 2.2 Fabrication of Three-Electrode System Using Laser-Induced Graphene

To provide a platform for electrochemical detection, LIG electrodes were fabricated by direct laser engraving of polyimide tape laminated onto cellulose paper using a CO_2_ laser system. The laser parameters, including power (3.6 W, 30%), scanning speed (25 mm/s), and resolution (400 DPI), were optimized to achieve uniform carbonization and formation of a porous graphene network. The laser engraving process locally converted the polyimide substrate to conductive graphene. A three-electrode configuration, consisting of a working electrode, counter electrode, and reference electrode, was directly patterned onto the polyimide substrate in a single step. Following laser processing, the electrodes were gently cleaned with compressed air to remove the residual debris.

### 2.3 Fabrication of Dual-Mode Paper-Based Microfluidic Analytical Device

Following LIG fabrication, tweezers are used to remove any remaining uncarbonized polyimide gently, leaving only electrodes deposited on the paper. The DM-μPAD was fabricated on LIG-deposited cellulose paper by defining hydrophilic channels and hydrophobic barriers using screen printing. The solution for screen printing contains MD700 (96 wt %) and Darocur 1173 (4 wt %), which is cured under nitrogen purging conditions with 365 nm UV light to form a clear hydrophobic barrier. During the screen-printing process, this resin mixture was selectively deposited onto the cellulose substrate through a mesh screen (100 mesh), allowing the monomer to fully penetrate the porous paper matrix. The fully cured resin forms dense, hydrophobic barriers that confine aqueous fluids within the unprinted, hydrophilic cellulose microfluidic channels via capillary-driven lateral flow (Figure 1).

**Figure 1:**
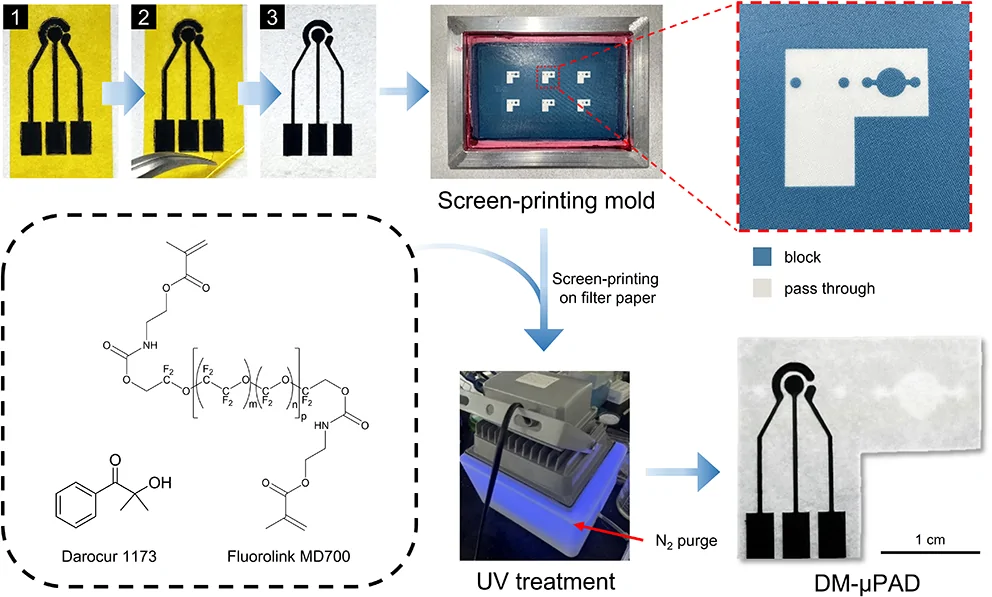
Fabrication process of the DM-μPAD by screen printing.

### 2.4 Preparation of Electrochemical Sensing Interface

To convert the hydrophobic surface of the fabricated LIG electrode into a hydrophilic surface, oxygen plasma pretreatment was performed. This involved a custom tape mask (3 M Scotch) to shield the reference and counter electrodes, while exposing only the working electrode. To maintain consistency during oxygen plasma treatment, the masking tape was positioned using the predefined electrode and substrate geometry as alignment references. The tape was applied in the same orientation and location relative to the electrode pattern for each device, ensuring that the exposed region subjected to oxygen plasma treatment remained consistent among samples. Therefore, the surface of the working electrode can be fully treated with oxygen plasma to facilitate subsequent surface modification. For electrochemical detection of glucose, the WE was modified with 1.25 μL/mm^2^ of GOx/chitosan solution (5 μL solution deposited on WE), which was prepared by dissolving 5 mg/mL of GOx and 3 mg/mL of chitosan in 0.5 mL DI water, followed by overnight incubation at 4 °C.^32^ Finally, the functionalized LIG electrodes were rinsed with DI water to remove unbound enzymes, dried under a nitrogen gas, and stored at 4 °C under dry conditions.

### 2.5 Dual-Mode Analytical Measurements (Electrochemical and Colorimetric)

Electrochemical measurements were performed using a potentiostat in a three-electrode configuration, where the LIG working electrode, counter electrode, and reference electrode were integrated on the paper substrate. CV and CA were employed to evaluate sensor performance. Glucose solutions with varying concentrations ranging from 0.01 to 3.0 mM were prepared in 0.1 M PBS. A fixed volume of 10 μL of sample was introduced into the microfluidic inlet, allowing it to flow to the sensing area. A 10 μL sample volume was selected to ensure adequate transport and repeatable contact with the sensing area for enhanced analytical reproducibility. All measurements were conducted at room temperature under ambient conditions. Electrochemical measurements were performed using the CA method. For colorimetric measurements, a defined volume of 1 μL of reagent mixture containing 18 U/mL GOx, 120 mU/mL HRP, and 2 mg/mL OPD was preloaded onto the colorimetric detection area. Smartphone images were acquired without a light-controlled enclosure. However, consistent lights, imaging distance, orientation, and smartphone settings were maintained throughout the measurements to minimize variability arising from ambient lighting. To record quantitative measurements, digital images of the colorimetric zones were captured using a smartphone camera at a fixed exposure distance under controlled, uniform illumination conditions. Quantitative colorimetric evaluation was subsequently performed by extracting the individual Red, Green, and Blue (RGB) intensity profiles from the captured images using ImageJ digital image processing software and the online analysis program Glucose Color Reader. For glucose detection, artificial sweat was used for practical applications. The preparation method was based on standard ISO 3160–2, using 20 g/L NaCl, 17.5 g/L NH_4_Cl, 5 g/L acetic acid, and 15 g/L lactic acid, and was adjusted with 0.1 M NaOH to pH 6.0.^33^ Subsequently, different concentrations of glucose were spiked into the artificial sweat for electrochemical and colorimetric sensing. All analytical measurements were performed using independently fabricated DM-μPAD units (*n* = 3) to ensure that reported error bars (Mean ± SD) represent complete device-to-device fabrication and measurement variability.

## 3 Results and Discussion

### 3.1 Design and Workflow of Integrated DM-μPAD

The DM-μPAD was designed as a self-contained, capillary-driven, analytical platform that integrates electrochemical and colorimetric sensing within a foldable two-layer paper microfluidic architecture (Scheme 1). A 3D microfluidic channel system was designed with hydrophobic barriers patterned on paper substrates via screen printing of MD700 to define the microfluidic channels. The first layer (Layer A) functions as the sample loading zone, directing fluid flow from a centralized injection point through the microchannels. The underlying layer is separated into distinct electrochemical (middle) and colorimetric sensing zones (side). The left well (Area 1) serves as the active colorimetric zone, while the right well (Area 2) acts as the control zone.

To fabricate the system, LIG electrodes were first patterned onto the paper using a CO_2_ laser, followed by hydrophobic screen printing to define the fluidic channels. The functional areas are then connected through a manual folding design (Figures 2A and S1). The cross-sectional diagram shows that the solution can reach the channel of Layer B through Layer A and contacts the LIG of Layer B (Figure 2B). The dual-mode sensing mechanism for glucose detection is illustrated in Figure 2C. Upon introducing the sample into the collection channel (Layer A), fluid first passes through the hydrophobic channel toward the detection zone and contacts the LIG electrode located in the second layer (Layer B). At the electrode interface, enzymatic oxidation of glucose by GOx, premodified on the surface, oxidizes the glucose to gluconolactone, generating H_2_O_2_. Under the application of an external potential, the H_2_O_2_ is oxidized to oxygen, releasing hydrogen ions and electrons, generating quantitative current response. As the sample continues to flow along the channels to the second layer, the glucose in the color-changing region is oxidized by GOx, generating H_2_O_2_ which oxidizes the colorless OPD via HRP catalysis to yellowish-brown 2,3-diaminophenazine (DAP). The adjacent control zone (Area 2), loaded only with GOx and HRP without OPD, serves to isolate background variations. The resulting optical color change was monitored and quantified via RGB image analysis using a smartphone. By integrating both sensing mechanisms within a single capillary-driven platform, the DM-μPAD provides simultaneous quantitative electrochemical measurement and visual colorimetric confirmation, improving analytical reliability through complementary dual-readout analysis.

**Figure 2:**
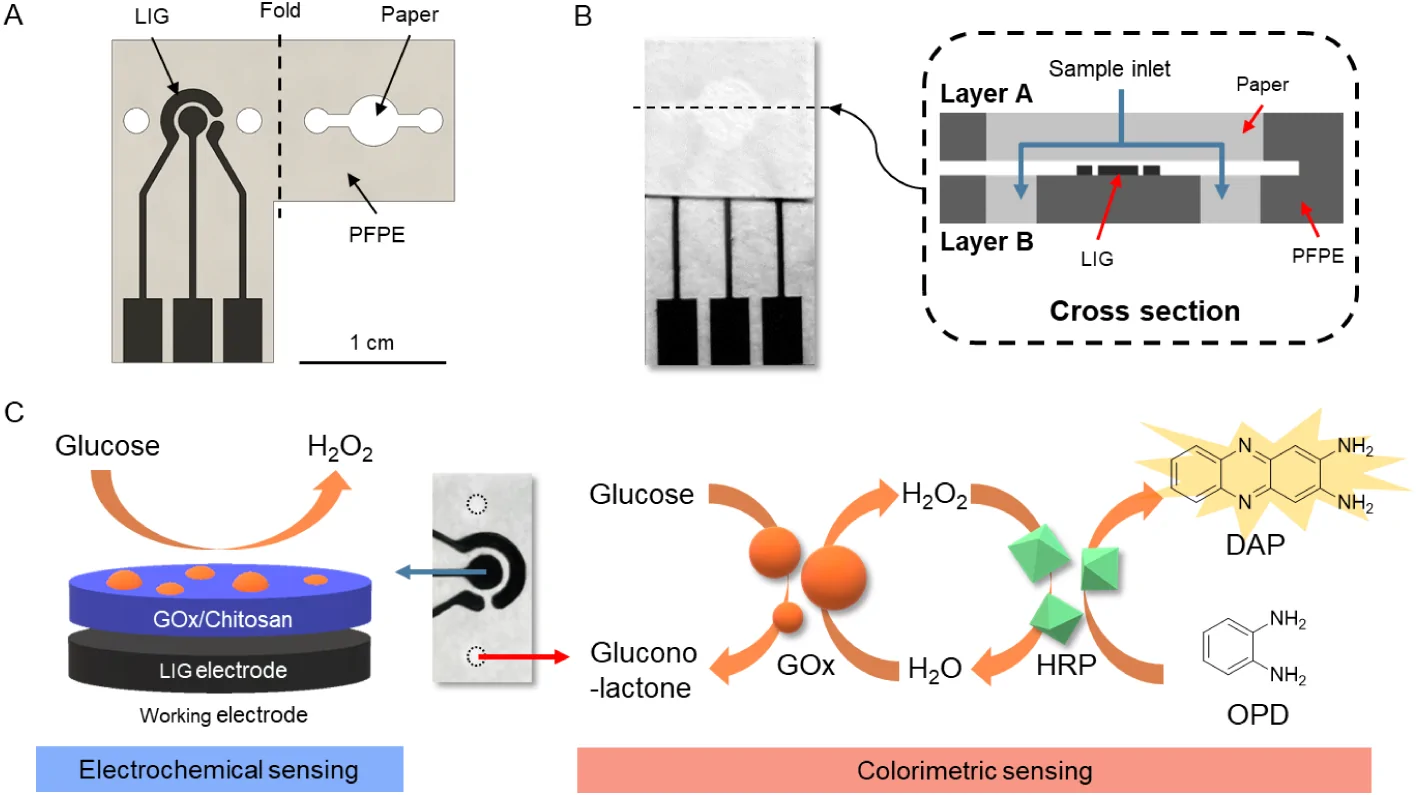
Design and functions of the DM-μPAD. (A) Structural design of DM-μPAD. (B) Cross-sectional illustration of sample flow and reagent interaction within the paper microfluidic network. (C) Mechanism of dual-mode glucose detection through electrochemical signal generation and colorimetric response.

Compared to traditional paper-based and dual-mode platforms, this approach offers distinct fabrication and operational advantages. While LIG electrodes can be formed directly on polyimide substrates,^34,35^ only a few materials possess the ability to directly generate LIGs. If other materials are required as substrates, electrode transfer methods are commonly used; however, these methods may affect electrode structure and detection performance.^36^ The DM-μPAD overcomes these limitations by utilizing polyimide tape, effectively combining the advantages of polyimide-derived LIGs with paper-based microfluidics. Furthermore, while multilayer 3D-folded paper devices efficiently utilize space, increasing the number of layers can lead to poor interlayer contact and unreliable sample flow.^37^ The DM-μPAD addresses this limitation through a two-layer architecture by custom 3D-printed clips, ensuring channel alignment, consistent capillary flow, and dual-mode integration. It is noted that the porous, 3D network of the LIG electrode functions as an electrochemically active interface rather than a primary fluidic channel. Sample transport is driven by the capillary pressure of the structured paper matrix in Layer A. Upon reaching the sensing zone, the solution travels to vertical fluidic contact with the LIG surface in Layer B. This architectural decoupling ensures that the high surface area and porous morphology of the LIG enhance enzymatic immobilization and charge-transfer kinetics without restricting microfluidic flow velocity.

### 3.2 Formation and Optimization of Laser-Induced Graphene Electrodes

To systematically determine the optimal laser processing parameters for the formation of conductive LIG using polyimide substrates, a 5 × 5 experimental parameter matrix was constructed by changing the laser power from 2.4 to 4.8 W (20 to 40%) and scan rates ranging from 15 to 35 mm/s (Figure 3A). This range was selected because LIG formation is governed by the localized photothermal conversion of the sp^3^-rich carbon network of PI to an electrically conductive sp^2^ graphitic network. Previous studies have shown that laser power, scan speed, and energy density strongly affect LIG morphology, graphitization quality, and electrical conductivity.^38,39^ Optical microscopy revealed that higher laser power combined with lower scan rate (**Area 1,** Figures 3A1 and S2A) resulted in more extensive carbonization of the cellulose fibers, forming pronounced vertically oriented, thread-like graphene structures, affecting charge-transfer efficiency.^40^ Conversely, lower laser power and higher scan rates delivered insufficient energy resulting in fewer graphene features to convert the polyimide substrate into LIG (**Area 3,** Figure 3A3). The optimized conditions were found where a more balanced laser power and scan rate were used (**Area 2,** Figures 3A2 and S2B). Together, these results indicate that there is a specific parameter window that is suitable for LIG fabrications.

**Figure 3:**
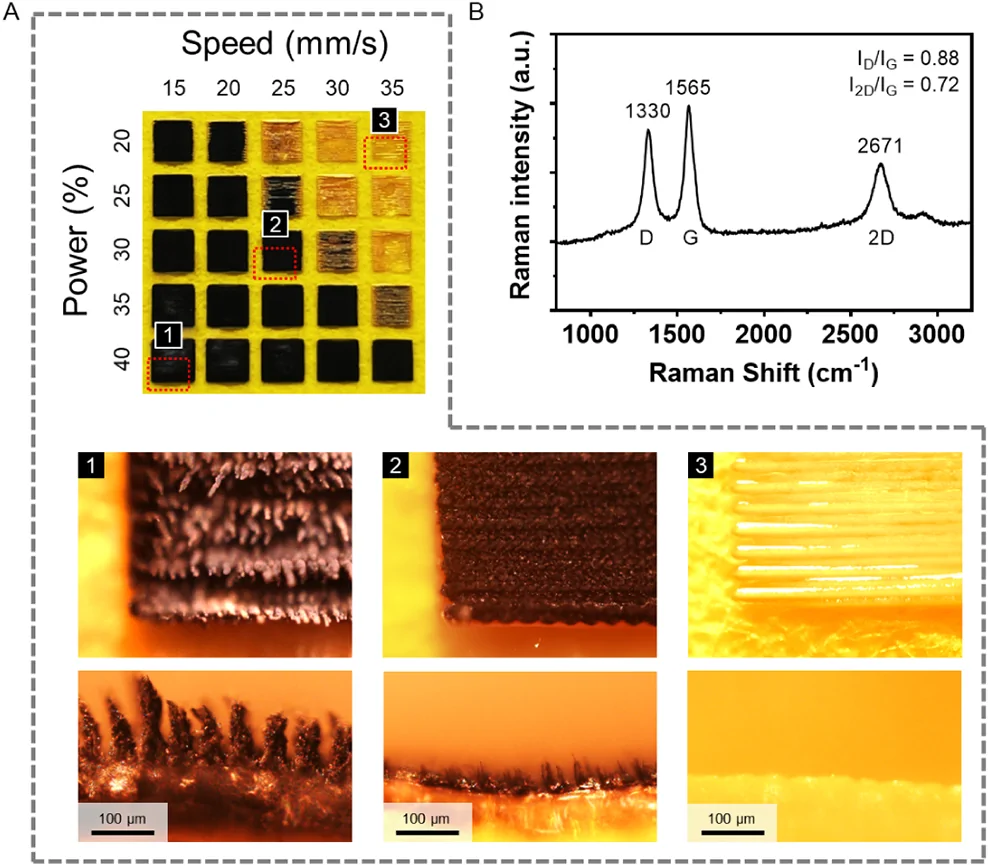
Characterization of LIG at different laser parameters. (A) Formation of LIG under different laser power and scanning speed conditions. The enlarged top view and cross-section from (A1) power 40% and speed 15 mm/s, (A2) power 30% and speed 25 mm/s, and (A3) power 20% and speed 35 mm/s under an optical microscope. (B) Raman spectra of the optimized LIG electrodes recorded using a 532 nm excitation laser, showing the characteristic D, G, and 2D bands of graphene.

To evaluate the LIG quality within this viable range, we performed Raman spectroscopy on samples synthesized under different parameters (Figure 3A), as it allows one to confirm the formation of graphene structures by observing D band, G band, and 2D band distributions. As a control, Raman characterization of the polyimide tape showed typical bands including C=O stretch at 1787 cm^–1^ (imide I), C–N–C axial vibration stretch at 1385 cm^–1^ (imide II), C–N–C transverse vibration stretch at 1115 cm^–1^ (imide III), C=C stretch at 1508 cm^–1^ in the phenyl ring, and aromatic imide at 1606 cm^–1^ (Figure S3).^41^ Meanwhile, the spectrum acquired on the area fabricated using laser power 3.6 W (30%) and speed 25 mm/s **(Area 2)** showed typical graphene characteristics with the defect-induced D band peak at 1330 cm^–1^, representing structural disorders; G band peak at 1565 cm^–1^ confirming the sp^2^-bonded hexagonal lattice; and 2D peak at 2671 cm^–1^ indicate graphene crystallization. The *I*_D_/*I*_G_ intensity ratio of 0.88 described the defect of graphene layers, which falls within the typical range of laser-induced electrodes; while excessive structural disorder can disrupt the continuous sp^2^ carbon network and increase electrical resistance, a moderate density of defects and edge sites can provide additional electrochemically active sites. Therefore, the electrical and electrochemical performance of LIG is determined by a balance between graphitic conductivity and defect/edge density.^42^ Additionally, the *I*_2D_/*I*_G_ intensity ratio of 0.72 indicates that the synthesized graphene layer consists of multilayers (Figure 3B).^43,44^

### 3.3 Characterization and Modification of the LIG Electrodes

To evaluate the electrochemical stability and reliability of the integrated LIG pseudoreference electrode, CV was conducted and compared directly against a commercial screen-printed carbon electrode (SPCE) featuring a standard Ag/AgCl reference electrode (Figure S4). The LIG pseudoreference electrode exhibited well-defined, reproducible redox peaks with stable anodic and cathodic peak potentials across various scans, demonstrating a minimal potential drift. While the open-circuit potential shifts slightly relative to Ag/AgCl due to the nature of carbon pseudoreference electrodes, the peak-to-peak separation and redox current responses remained consistent and comparable to those of the SPCEs. These results confirm that the LIG three-electrode configuration provides sufficient potential stability and operational reproducibility for microfluidic biosensing without requiring additional Ag/AgCl-deposition steps.

To evaluate the charge-transfer properties and effective surface areas of the fabricated platforms, CV was performed using a 5 mM [Fe­(CN)_6_]^3–/4–^ redox probe in a 0.1 M KCl supporting electrolyte at varying scan rates ranging from 10 to 150 mV/s. The resulting voltammograms demonstrated that both anodic and cathodic peak current intensities were linear with the square root of the scan rate, confirming a diffusion-controlled electrochemical reaction pathway at the LIG interface (Figure 4A and 4B). Additionally, electrodes with different widths (0.5, 1.0, and 1.5 mm) were evaluated for comparative testing. The results showed no significant differences in charge-transfer efficiency, and a 0.5 mm width was selected for subsequent experiments to minimize both fabrication time and energy consumption (Figures 4A and S5).

**Figure 4:**
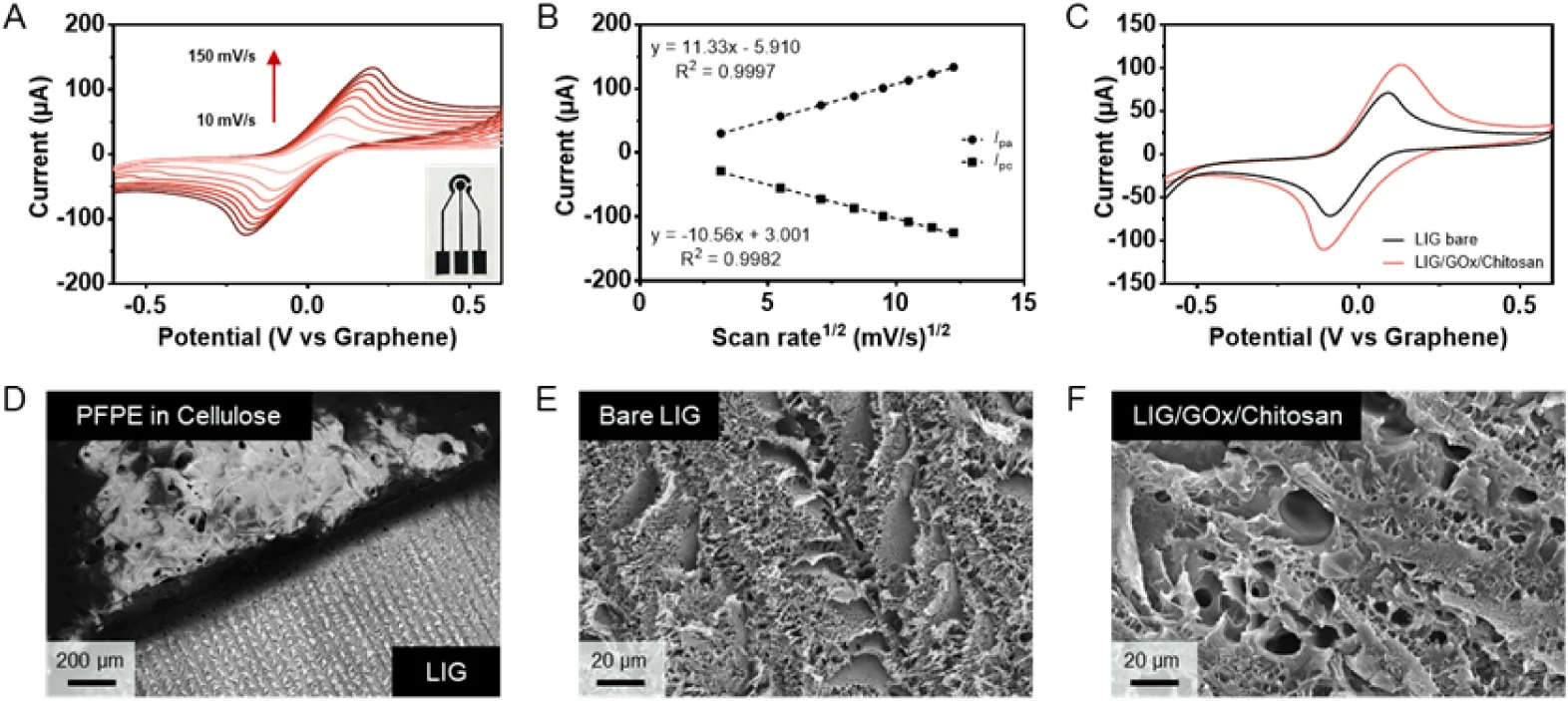
Electrochemical and morphological characterization of the LIG electrodes. (A) Cyclic voltammograms of the LIG electrode recorded in 5 mM [Fe­(CN)_6_]^3–/4–^ containing 0.1 M KCl at different scan rates. (B) Linear relationship between the anodic/cathodic peak currents and the square root of the scan rate. (C) CV responses of bare LIG and GOx/chitosan-modified LIG electrodes in 5 mM [Fe­(CN)_6_]^3–/4–^ containing 0.1 M KCl. SEM images of (D) the interface between the paper substrate and LIG region, (E) the bare LIG electrode surface, and (F) the GOx/chitosan-modified LIG electrode.

Laser engraving of cellulose paper resulted in the in situ formation of a porous graphene network, driven by localized photothermal carbonization. The resulting LIG structure exhibited a hierarchical porous morphology, which significantly increases the electrochemically active surface area and facilitates electrolyte diffusion. Moreover, the interconnected graphene framework enhances electrical conductivity and accelerates charge-transfer kinetics, which are critical for high-performance electrochemical sensing. Compared to conventional carbon-based electrodes, LIG provides a unique combination of scalability, patternability, and high electrochemical activity. In comparison with bare LIG electrodes, GOx/chitosan-modified LIG exhibited higher redox peak currents toward ferri/ferrocyanide [Fe­(CN)_6_]^3–/4–^, indicating enhanced electroactive surface area and efficient electron transfer capability (Figure 4C). Moreover, the SEM images revealed that the modification introduced a polymer-like thin structure on the LIG surface, confirming successful immobilization of GOx/chitosan while maintaining the porous conductive network of LIG (Figures 4D, 4E, 4F, and S6).

### 3.4 Optimization of Microfluidic Barriers for the DM-μPAD

To ensure robust fluidic flow within the paper channels, we tested MD700, PDMS, crayon, and paraffin wax as materials to fabricate hydrophobic barriers and determined their hydrophobicity by measuring contact angles (Figure S7A). The results showed that the paper surface treated with MD700 exhibited the highest contact angle (128.4 ± 0.2°), demonstrating superior hydrophobic barrier properties compared to PDMS, crayon, and paraffin wax. This enhanced hydrophobicity effectively confines the aqueous sample within the defined channels, preventing solution leakage and crosstalk between different areas. Using MD700 as a hydrophobic barrier, the nitrogen exposure time was optimized, and it was found that a curing time of only 30 s was sufficient to form flow channels on the cellulose paper (Figure S7B).

To improve the efficiency and optical intensity of colorimetric assays, the concentrations of the preimmobilized enzymatic reagents (GOx, HRP, and OPD) were subsequently optimized. Spectrophotometric analysis revealed that the peak absorbance at 420 nm, corresponding to the generation of the DAP product, was maximized at enzyme loadings of 18 U/mL GOx and 120 mU/mL HRP (Figure S8A and S8B).

However, excessive concentrations of the OPD substrate led to nonspecific autoxidation. This autoxidation occurs even in the absence of HRP or H_2_O_2_, creating elevated background signals that significantly narrowed the linear detection range of the platform. Consequently, a baseline concentration of 2 mg/mL OPD was selected to successfully suppress nonspecific background interference while maintaining a distinct, visible colorimetric signal for accurate visual and RGB quantification (Figure S8C).

To determine the minimum sample volume required for DM-μPAD detection, we performed tests in 1 μL increments using a blue dye for easier visual observation. The results showed that 3 μL of blue dye was added before the blue color was observed in the channel on the back of the device, thus demonstrating that at least 3 μL of sample is required for successful detection (Figure S9).

### 3.5 Integrated Electrochemical Sensing Module

The DM-μPAD architecture enables a single sample to be passively distributed to electrochemical and colorimetric sensing regions, allowing simultaneous quantitative and visual analysis without additional sample handling. To ensure a stable connection across the paper-based microfluidic channels, we designed a custom 3D-printed part to clamp DM-μPAD without affecting sample injection and colorimetric visualization. This strategy prevents unnecessary movement or displacement of the paper substrates during sample loading, which could otherwise lead to misalignment of the fluidic channels and interfere with the accuracy of the test results (Figure 5A).

**Figure 5:**
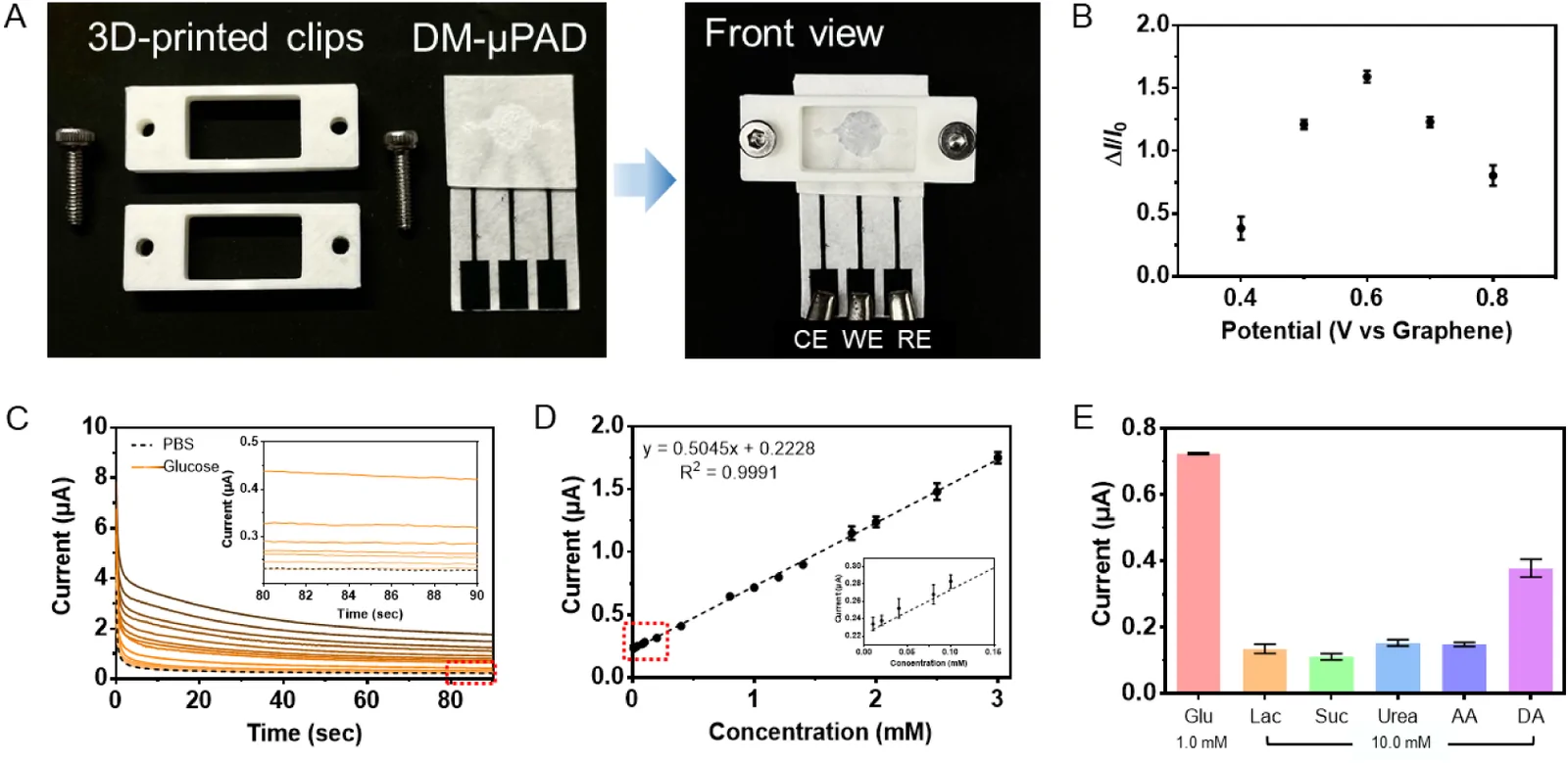
Electrochemical detection of glucose. (A) Optical image of the front view of the DM-μPAD. (B) Current response of the LIG sensor toward 2 mM glucose at different applied potentials for optimization. (C) Chronoamperometric current responses of the GOx/chitosan-modified LIG sensor toward varying glucose concentrations ranging from 0.01 to 3.0 mM. (D) Resulting calibration curve plotting the steady-state current response against glucose concentration. (E) Selectivity testing of the electrochemical glucose sensor challenged with common physiological interferents. Error bars represent standard deviation (*n* = 3).

To determine the optimal operating potential for CA glucose detection, the change in current response was evaluated across different applied potentials using a fixed glucose concentration of 2 mM. The maximum current change (Δ*I*) was observed at approximately 0.6 V. Therefore, this potential was subsequently selected as the operating potential for all the following CA measurements. This electrochemical behavior is directly related to the oxidation of the enzymatically generated H_2_O_2_. At potentials below 0.6 V, the driving force is insufficient to efficiently oxidize H_2_O_2_, resulting in a decrease in current signals. However, when the potential is too high, it easily leads to an increase in background current and may even cause other interfering substances to be oxidized and can compromise the sensing selectivity. Therefore, 0.6 V was considered the optimal condition balancing oxidation efficiency and signal stability (Figure 5B).

Upon introducing varying concentrations of glucose under the optimized conditions, the current decreased rapidly and reached a steady state within 90 s. The magnitude of the current response correlated directly with the target glucose concentration. The reason is that the volume of H_2_O_2_ produced by GOx catalysis increases with higher glucose concentrations, and therefore the oxidation current also increases at the electrode surface; a control experiment verified that bare LIG exhibits no change in oxidative current in response to glucose (Figure S10). Based on the resulting calibration curve, the electrochemical sensing mode of the DM-μPAD delivered a broad linear detection range from 0.01 to 3.0 mM. The limit of detection (LOD) was found to be 9.23 μM utilizing the standard 3σ/S curve (where σ represents the standard deviation of the blank signal and S denotes the sensitivity slope of the calibration curve) (Figure 5C and 5D). To evaluate the performance of the DM-μPAD, its sensing capability was compared with previously reported electrochemical and colorimetric glucose sensors (Table S1). For electrochemical detection, our platform achieves a notably broader linear range (0.01–3.0 mM) than several existing paper- and LIG-based sensors, such as those utilizing PEDOT:PSS/Ti_3_C_2_/MWCNT, PB/GOx, or Pt-NPs/Zein/Gel-GOx, while maintaining a comparable LOD of 9.23 μM.

To further validate the selectivity of DM-μPAD, the current responses of several common physiological interfering agents were evaluated and compared, including lactose, sucrose, urea, AA, and DA. Using 1.0 mM glucose as the baseline, all interfering substances were introduced at a concentration of 10.0 mM to verify the anti-interference capability of the electrochemical mode. The results showed that the signal generated by glucose was significantly higher than those of the other interfering agents, with only DA producing a notable current response. This response occurs because DA readily undergoes direct oxidation at a working potential of +0.6 V.^45^ However, even under these extremely high concentrations of DA, which is substantially higher than physiological dopamine levels, a difference in current between DA and glucose was observed, indicating that DM-μPAD possesses good anti-interference capabilities. This selectivity is fundamentally attributed to the stringent biocatalytic specificity of the immobilized GOx toward glucose molecules (Figure 5E).

Selectivity was further evaluated by measuring the current response of glucose in the direct presence of coexisting physiological sweat interferents (Figure S11). While neutral and nonelectroactive interferents caused negligible current variations, the electroactive species DA exhibited a slight anodic current when mixed with glucose. Operating at an applied potential of +0.6 V vs graphene is required to drive the direct electrochemical oxidation of the enzymatically produced H_2_O_2_. The combination of the biocatalytic turnover rate of the immobilized GOx and the large electrochemically active surface area of the LIG matrix ensures that the glucose-specific H_2_O_2_ signal predominates, thereby maintaining sufficient analytical selectivity for practical sweat analyses.

Next, the storage stability of the enzyme-modified DM-μPAD was evaluated over a 21 day period to determine its viability for practical, long-term biosensing of glucose. The relative response intensity of the sensor was monitored at set intervals under controlled storage conditions (4 °C). The DM-μPAD demonstrated good stability, retaining 88.05% of its signal intensity even after 21 days of storage. These results confirm that the fabricated dual-mode platform possesses sufficient shelf life for practical diagnostic applications (Figure S12).

### 3.6 Integrated Colorimetric Module and RGB Analysis

In addition to electrochemical detection, DM-μPAD incorporates visual detection mode utilizing colorimetric mode to provide a simple and convenient validation method. While its sensitivity might be lower than that of the electrochemical mode, it offers reliable monitoring of glucose concentration. The sensing mechanism is driven by an enzymatic cascade. GOx oxidizes glucose to generate H_2_O_2_, which, in the presence of HRP catalysis, oxidizes the colorless OPD substrate into a yellowish-brown DAP product. UV–vis spectrophotometric profiles confirm a characteristic absorption maximum at 420 nm corresponding to the oxidized DAP product (Figure 6A). To demonstrate the specificity of this colorimetric reaction, multiple control experiments were conducted. As illustrated in Figure S13, the absorbance peak at 420 nm was exclusively observed when GOx, HRP, OPD, and glucose were present in the reaction mixture. The control groups lacking any of these components exhibited no significant absorption, validating the operational feasibility of the colorimetric detection system.

**Figure 6:**
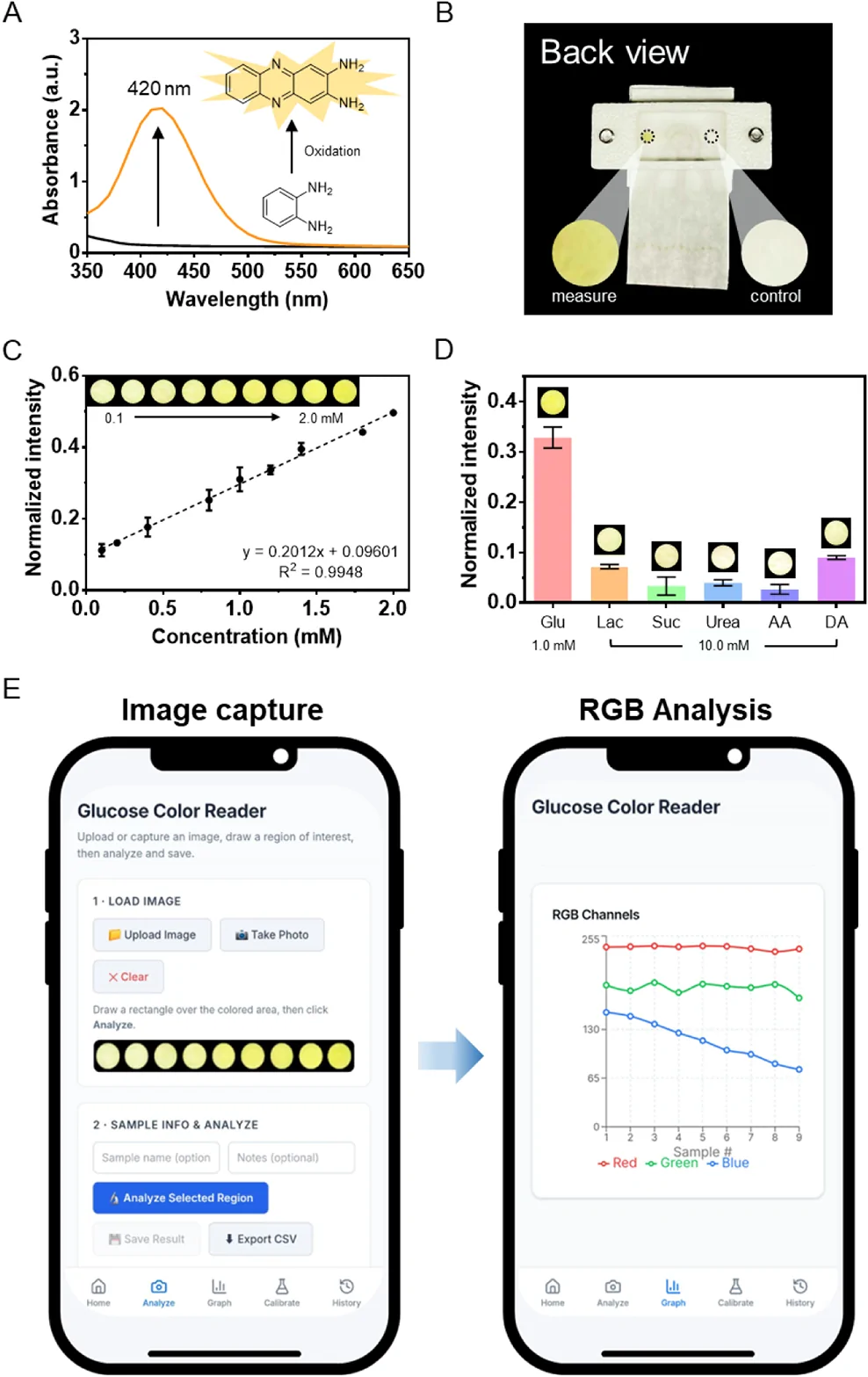
Colorimetric detection of glucose. (A) UV–vis absorption spectra and corresponding color change arising from the oxidation of OPD to DAP. (B) Optical image of the reverse view of the DM-μPAD showing the dual-channel design. (C) Calibration curve plotting the normalized color response against glucose concentration. (D) Selectivity testing of the colorimetric assay in the presence of common sweat interferents. (E) Schematic representation or workflow of the smartphone-based colorimetric image analysis. Error bars represent standard deviation (*n* = 3).

The DM-μPAD features two parallel circular microdetection areas on its reverse side: a primary measurement area and an adjacent reference control area. Upon sample incubation, the measurement area develops a distinct yellowish-brown color via oxidation of glucose, whereas the control area retains its original milky white appearance. This dual-channel design effectively eliminates background interference and improves overall detection accuracy (Figure 6B). Quantitatively, as glucose concentration increases, the yellowish-brown color in the measurement area visibly deepens. By extracting and normalizing the optical profiles using the blue intensity from RGB image analysis, a linear range of 0.1 to 2.0 mM was established, yielding an optical limit of detection of 57.9 μM based on the standard 3σ/Slope method (3σ/Slope of the calibration curve) (Figure 6C). Compared to previous studies, the colorimetric mode provides a wide linear range of 0.1–2.0 mM, outperforming several paper-based alternatives (Table S1). While some platforms offer slightly lower LODs using more complex materials or instrumentation, our dual-mode DM-μPAD offers a practical balance of a broad dynamic linear range, low detection limits, and a low-cost paper/LIG architecture suitable for point-of-care sweat monitoring.

To evaluate the selectivity of the colorimetric mode, the detection platform was exposed to common sweat interfering substances, including lactose, sucrose, urea, AA, and DA at concentrations as high as 10.0 mM against a 1.0 mM glucose baseline. The results showed that, although the concentrations of the interfering substances were much higher than those of glucose, the normalized intensity and corresponding color development were extremely low. Only glucose elicited a significantly strong colorimetric signal, demonstrating that the detection system has high specificity and selectivity for glucose and resists interference from common chemical species found in sweat or other biological fluids (Figure 6D).

To enable on-site, in situ glucose monitoring, the DM-μPAD platform integrates smartphone-based colorimetric analysis using a colorimeter application (Figure 6E). This system transforms captured images into digital Red, Green, and Blue (RGB) values, with each channel providing a density range of 0 to 255 to quantify even small color shifts.^46^ Specifically, the blue color ratio decreases as the concentration increases, correlating directly with the oxidation of OPD. This smartphone-based approach provides a low-cost, user-friendly, and precise platform for rapid, point-of-care glucose quantification. We also developed a simple 3D-printed portable detection system to enable colorimetric and electrochemical analysis using the DM-μPAD (Supporting Information and Figures S14–S16). The system can accommodate both sensing modalities within a single platform. The results demonstrate that the reader can reliably distinguish multiple concentration ranges of the paper sensor and provide immediate on-screen classification.

### 3.7 Detection of Glucose in Artificial Sweat

To assess the practical utility and potential for future applications of the DM-μPAD, the dual-mode platform was used for glucose quantification in artificial sweat and PBS solutions. Compared to the PBS solution, real biological fluids typically contain complex amounts of salts, organic molecules, and proteins, thus easily leading to matrix effects that can affect sensing accuracy. To evaluate the platform performance against these factors, target glucose was introduced at concentrations of 0.3 mM, 0.5 mM, 0.9 mM, 1.3 mM, and 1.7 mM. The results show that DM-μPAD maintained stable current and color response that increased with concentration across all tested biological fluids in both electrochemical and colorimetric modes (Tables 1 and 2). This confirms that the designed DM-μPAD effectively mitigates interference and signal distortion, highlighting its potential for noninvasive portable biosensing applications. However, future testing using real human sweat samples will still be necessary to validate its clinical application for POCT.

**Table 1:** Detection of Glucose in Artificial Sweat with DM-μPAD

Mode	Added conc. (mM)	Detected conc. (mM)	Recovery (%)	RSD (%)
Electrochemical	0.3	0.31	102.9	8.6
	0.5	0.51	102.8	1.6
	0.9	0.96	107.3	4.3
	1.3	1.24	95.2	4.8
	1.7	1.67	98.3	1.3
Colorimetric	0.3	0.31	102.3	5.4
	0.5	0.53	106.4	7.9
	0.9	0.94	104.3	4.6
	1.3	1.29	99.2	8.2
	1.7	1.68	98.6	1.5

**Table 2:** Detection of Glucose in PBS with DM-μPAD

Mode	Added conc. (mM)	Detected conc. (mM)	Recovery (%)	RSD (%)
Electrochemical	0.3	0.30	100.6	5.3
	0.5	0.49	98.4	3.5
	0.9	0.94	103.9	1.0
	1.3	1.28	98.5	2.6
	1.7	1.69	99.2	1.1
Colorimetric	0.3	0.30	100.2	6.6
	0.5	0.49	97.4	2.9
	0.9	0.92	102.4	2.6
	1.3	1.29	99.4	2.4
	1.7	1.68	98.9	3.4

## 4 Conclusion

The DM-μPAD was successfully developed by integrating LIG-based electrochemical sensing with enzyme-mediated colorimetric detection into a single capillary-driven paper platform. The fabrication strategy combines one-step CO_2_ laser patterning of conductive LIG electrodes with screen-printed hydrophobic microfluidic channels eliminating the need for flame retardants and electrode transfer processes, thereby simplifying device fabrication. The integrated architecture enables passive fluid distribution to both sensing modules, allowing the simultaneous electrochemical quantification and visual colorimetric confirmation from a single sample. When evaluated for glucose detection, the electrochemical mode offers high sensitivity and a broad linear detection range of 0.01 to 3.0 mM, while the colorimetric mode enables visual detection over the 0.1 to 2.0 mM range and serves as an auxiliary verification method. The study further demonstrates that DM-μPAD could provide high performance in simulated sweat environments, making it a promising and viable platform for future applications in noninvasive portable health monitoring, POCT, and low-cost home medical diagnostics.

## Supplementary Material



## Notes

Design and principle of a photodiode-based detection system, fabrication and surface modification process of the DM-μPAD, Raman spectra, CV response of LIG with different widths, hydrophobicity test using contact angle, optimization of enzymatic reagent concentrations, custom 3D-printed device, and tables and figures (PDF)
