# Supplementary material for: Laser-Induced Graphene Paper-Based Microfluidic Platform for Dual-Mode Electrochemical and Colorimetric Detection of Glucose
Source: ACS Omega. 2026 Oct 24;11(44):69117–26. doi: 10.1021/acsomega.6c09156 (PMC13756711; doi:10.1021/acsomega.6c09156)
Supplement: Supplementary file 1 [file ao6c09156_si_001.pdf]

Supporting information

Laser-Induced Graphene Paper Microfluidic Platform for Dual-Mode Electrochemical and Colorimetric Detection of Glucose

*Che-Liang Yeh, Syed Atif Ali, Wen-Ti Wu and Hsiung-Lin Tu**

Institute of Chemistry, Academia Sinica, Nankang, Taipei 11529, Taiwan

**Email: hltu@gate.sinica.edu.tw*

Figure S1-S17
Table S1

Experimental methods

Design and Principle of Photodiode Based Detection System

An Arduino-based detection system was developed to quantify the colorimetric response of the paper sensor using a photodiode. The photodiode output was continuously acquired through the Arduino analog input and transmitted to a custom processing interface. The Arduino code was modified to convert the measured light intensity into glucose concentration using the calibration equation obtained from the intensity-concentration curve. As a result, the system was capable of directly displaying glucose concentration values corresponding to the color change of the paper sensor in real time, eliminating the need for manual image analysis **(Figure S9)**. A photodiode module was interfaced with an Arduino microcontroller for colorimetric signal acquisition. The sensor was powered through the Arduino supply pins, with VIN connected to the 5 V output and GND connected to the common ground. Communication between the photodiode module and Arduino was established through the SDA and SCL pins. The integrated LED of the sensor provided consistent illumination of the colorimetric paper strip during measurements. The reflected light intensity from the paper sensor was detected by the photodiode and transmitted to the Arduino, where the signal was processed and sent to a custom Processing-based graphical user interface for real-time visualization and concentration analysis.

To further visually increase the functionality of the reader, the Arduino program was modified to provide quantitative glucose measurements. A calibration curve was first established by correlating the RGB intensity values obtained from ImageJ analysis with known glucose concentrations. The resulting regression equation was then incorporated into the Arduino code, enabling real-time conversion of photodiode signals into glucose concentration values. When an unknown sample was introduced, the system automatically calculated and displayed the corresponding glucose concentration (mg mL^{-1}) on the screen. It should be noted that quantitative colorimetric concentration determination reported in the main manuscript was conducted using smartphone image acquisition and ImageJ software processing. The TCS34725 module was implemented as a proof-of-concept platform for rapid, semi-quantitative color classification (identifying discrete concentration thresholds based on raw channel intensity) rather than continuous quantitative concentration estimation. Consequently, smartphone-based calibration curves were not directly transferred to the TCS34725 hardware module, maintaining a clear distinction between quantitative image analysis and hardware-based threshold classification.

Portable Photodiode-Based Detection of Colorimetric Glucose Assays

A custom 3D-printed sensing chamber was designed and fabricated to enable dual-mode analysis of paper-based sensors. The chamber incorporates an integrated optical compartment containing the photodiode for colorimetric measurements. In addition, an upper insertion slot was designed to accommodate three copper electrode contacts, allowing direct electrical interfacing with paper-based LIG electrochemical sensors. This integrated platform enables both optical and electrochemical signal acquisition using a single compact and portable device, facilitating versatile point-of-care sensing applications. The Arduino-photodiode platform was used to monitor the colorimetric paper sensor and display the corresponding concentration in real time **(Figure S10)**. Different paper samples produced distinct photodiode intensity values, which were processed by the Arduino and visualized through a custom Processing interface. The interface successfully differentiated the sensor responses into predefined categories,

including “No Sample,” “Negative,” and concentration levels labeled Conc A-Conc F **(Figure S11**).

The results for this interface are shown in **Figure S11**. In the absence of a sample, the system reported “No Sample”. When a negative sample was inserted, the interface showed the “Negative” category. Samples with increasing color intensity were classified as Conc A, Conc B, Conc C, Conc D, Conc E, and Conc F. The measured RGB values displayed at the top of the interface changed systematically with the sensor color, indicating that the photodiode signal was sensitive to variations in the colorimetric response. These results demonstrate that the Arduino-based reader can reliably distinguish multiple concentration ranges of the paper sensor and provide immediate on-screen classification without requiring manual image analysis. The platform therefore offers a simple and low-cost approach for portable colorimetric sensing.

Dual-Mode Sensing Performance

To validate the cross-verification capability of the dual-mode platform, identical glucose samples were simultaneously measured via both electrochemical and colorimetric channels **(Figure S17)**. A Bland–Altman analysis demonstrated close agreement between the two detection modalities, exhibiting a minimal mean bias and narrow 95% limits of agreement, with measured concentrations falling within acceptable statistical limit. Additionally, the reproducibility of sample distribution between the two sensing regions was verified by evaluating the chip-to-chip signal variability across multiple fabricated devices (n = 3).

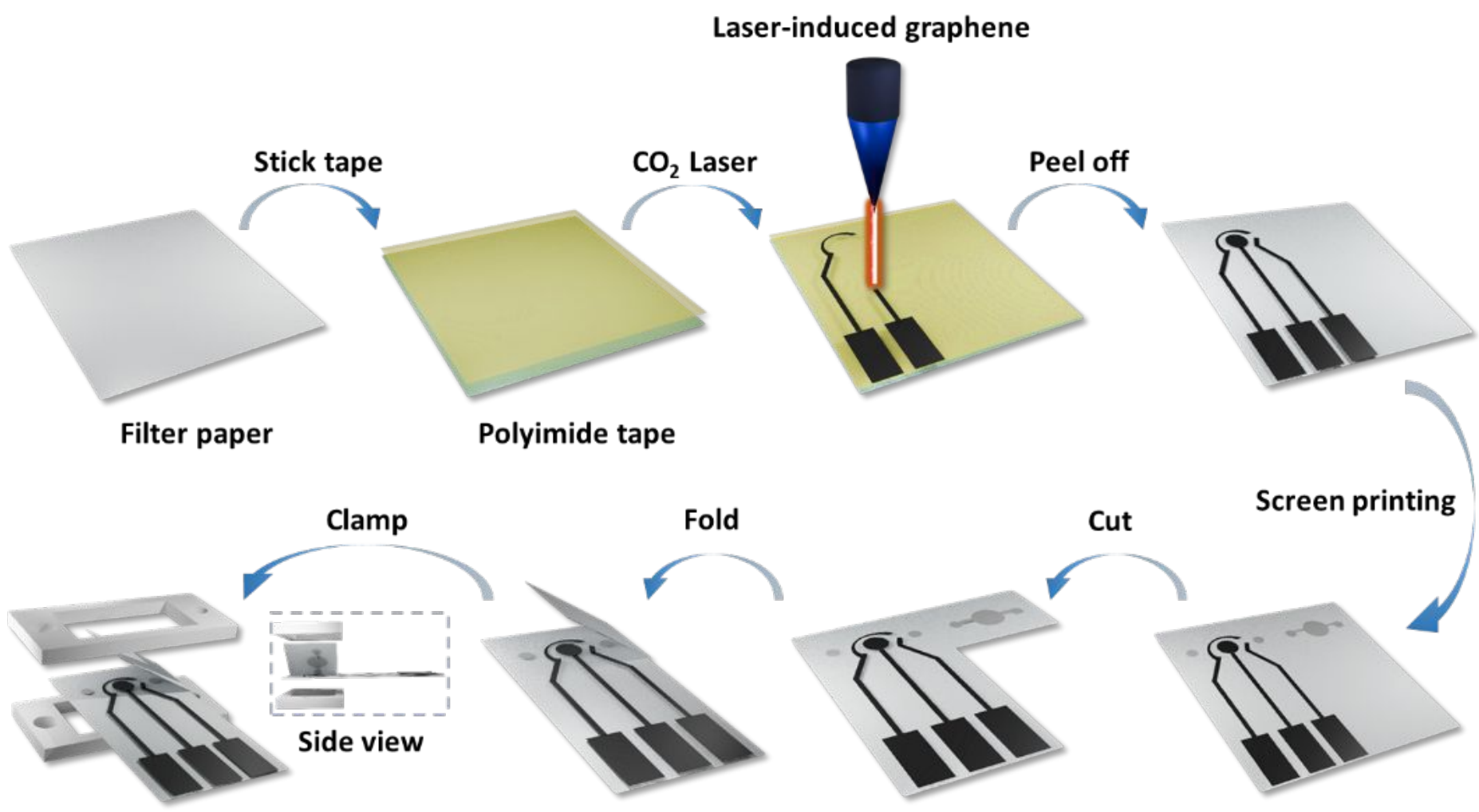

Figure S1. Fabrication and surface modification process of the DM-μPAD through laser-induced graphene formation on the PI/paper substrate.

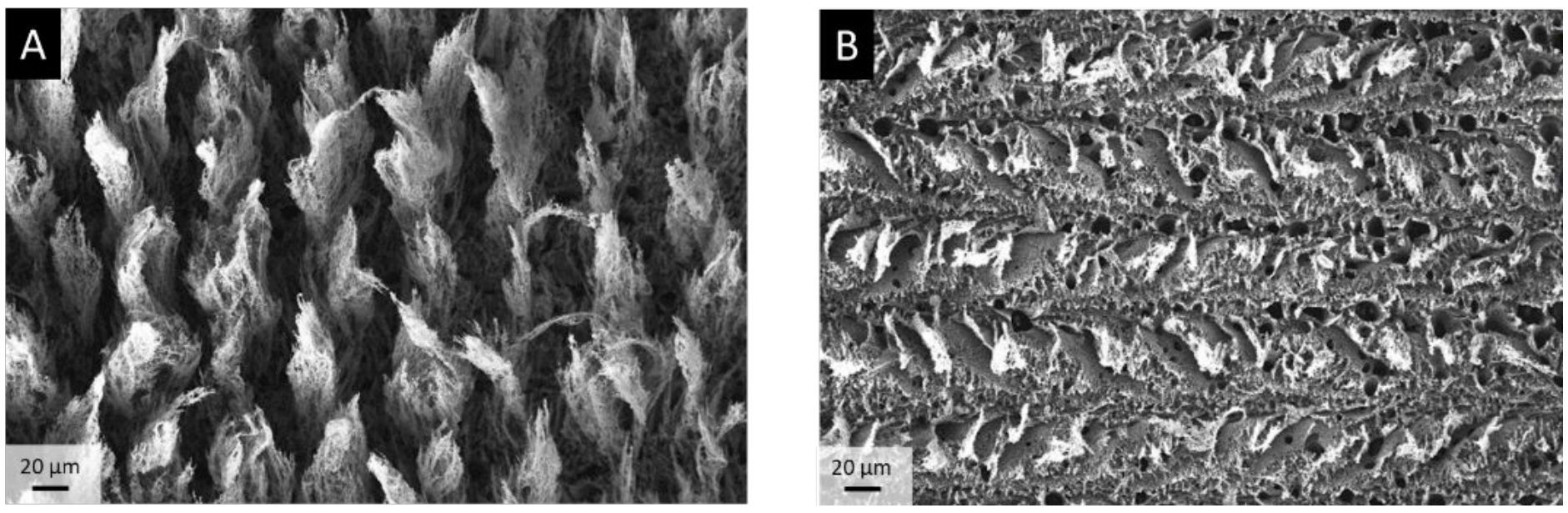

Figure S2. Higher magnifications SEM images in characterization of LIG at different laser parameters. (A) Power 40 % and speed 15 mm/s. (B) Power 30 % and speed 25 mm/s.

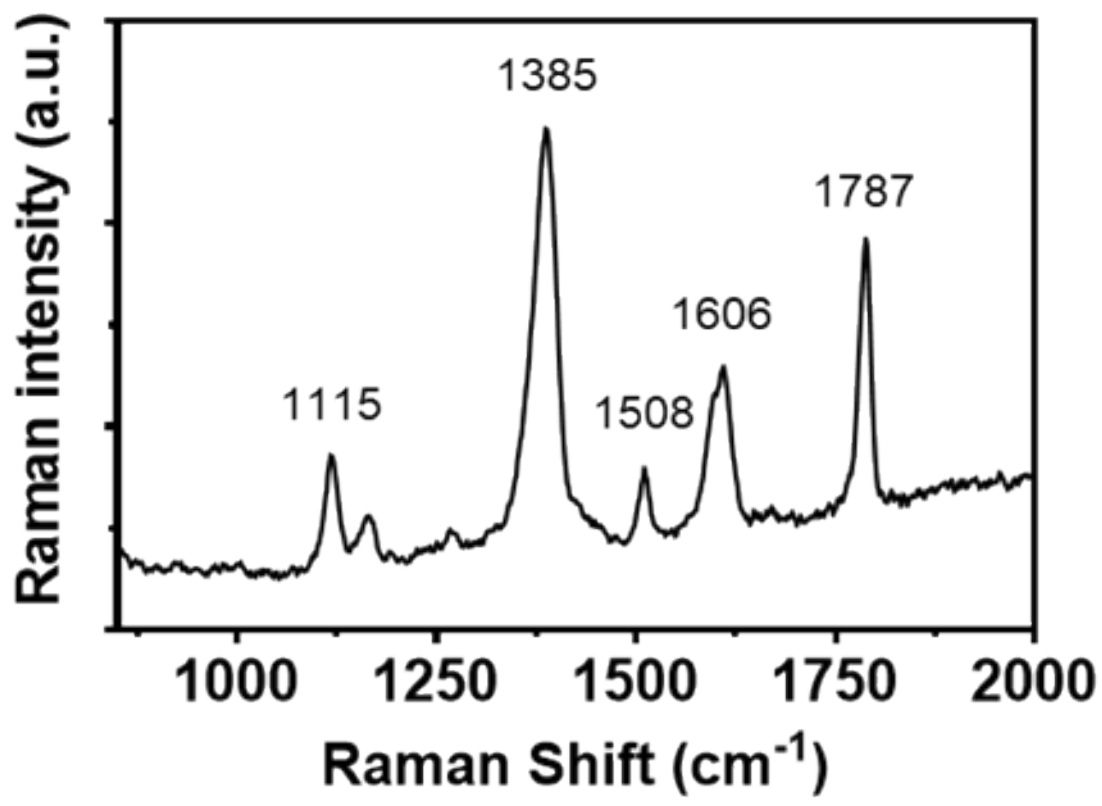

Figure S3. Raman spectrum obtained from fabrication of laser-induced graphene on polyimide tape.

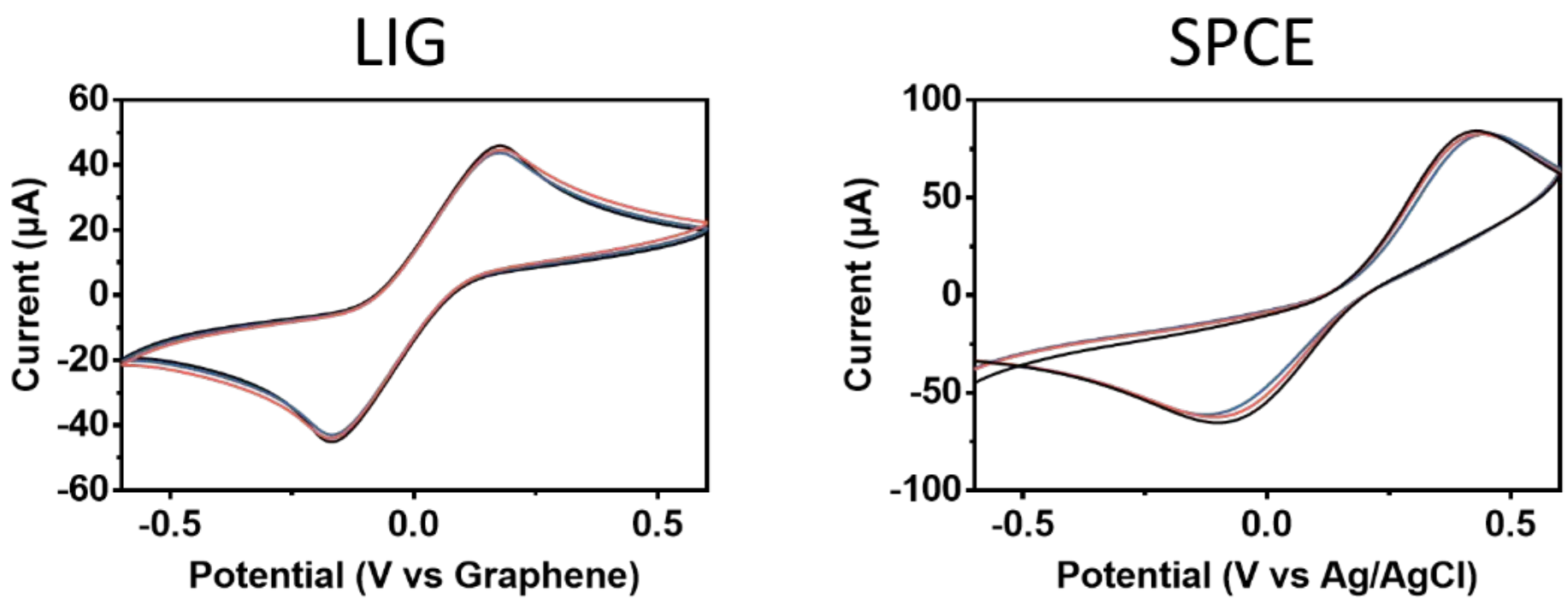

Figure S4. Cyclic voltammograms of the LIG and SPCE electrodes recorded in 5 mM $[Fe(CN)_6]^{3-/4-}$ containing 0.1 M KCl of three independent measurements (n = 3).

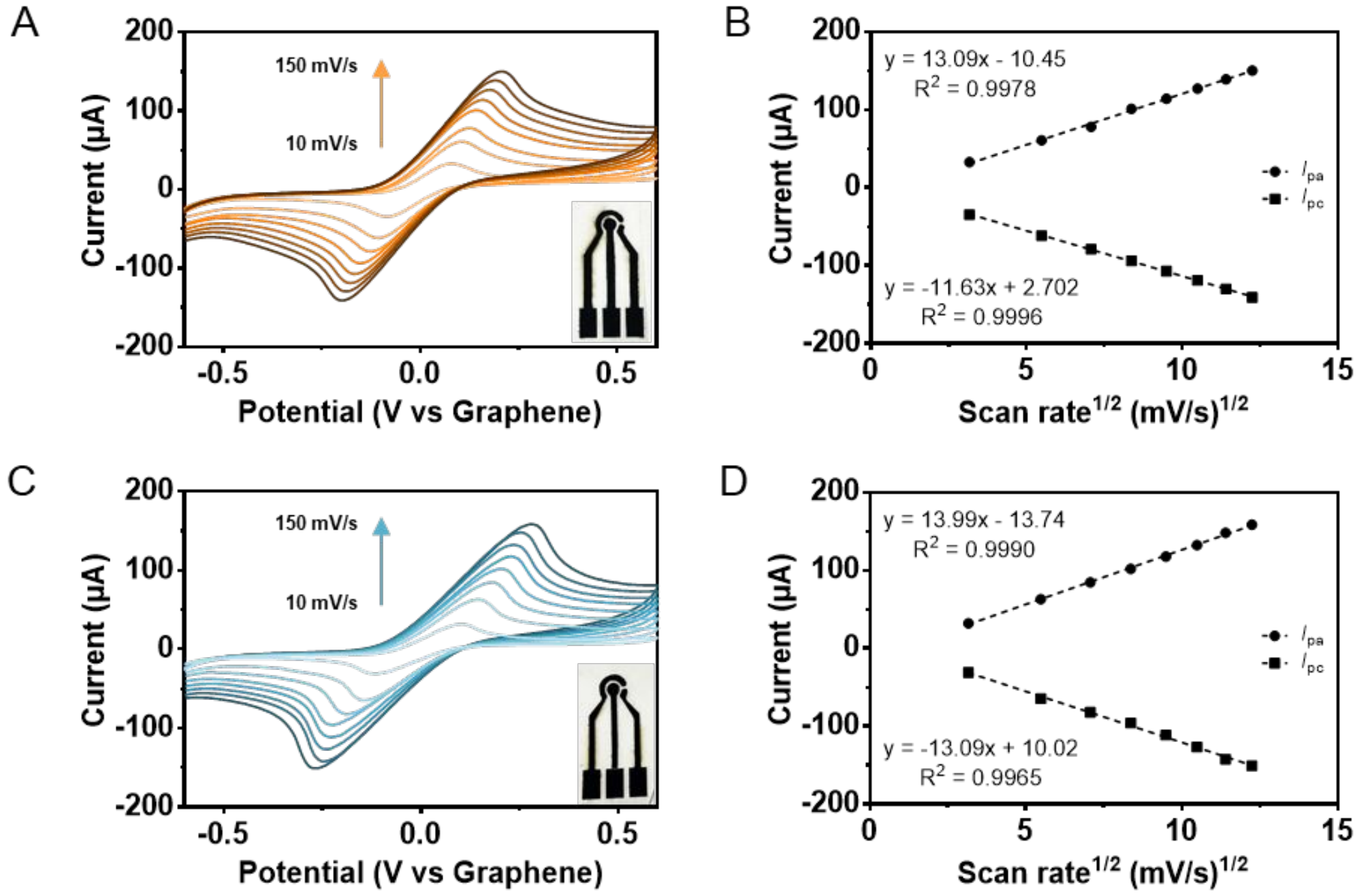

Figure S5. CV response of LIG with different widths. (A) Width of LIG electrodes: 1.5 mm. (B) The plot of peak current versus square root of corresponding scan rate with 1.5 mm electrodes. (C) Width of LIG electrodes: 1.0 mm. (D) The plot of peak current versus square root of corresponding scan rate with 1.0 mm electrodes.

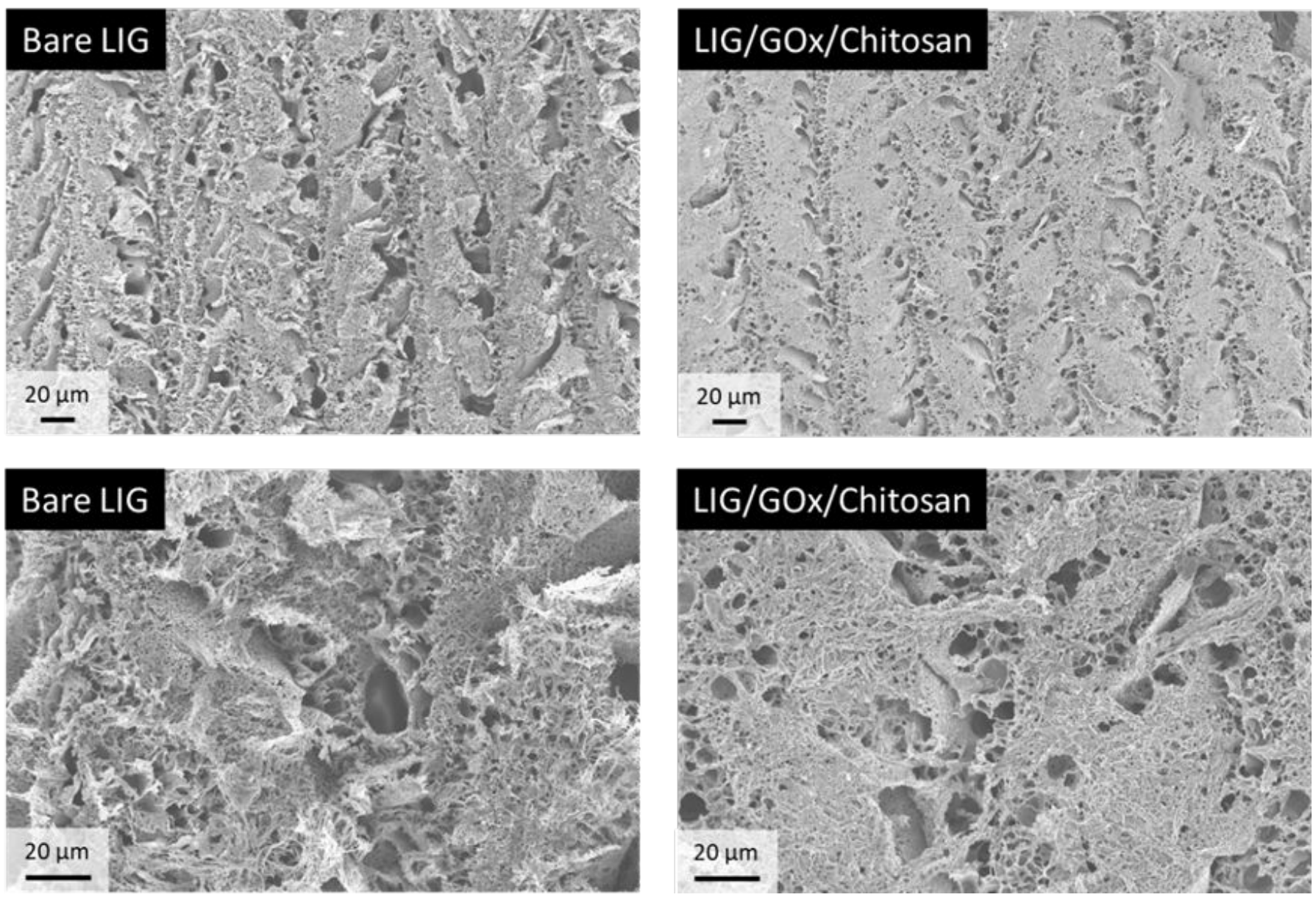

Figure S6. Higher magnifications SEM images in characterization of LIG modification.

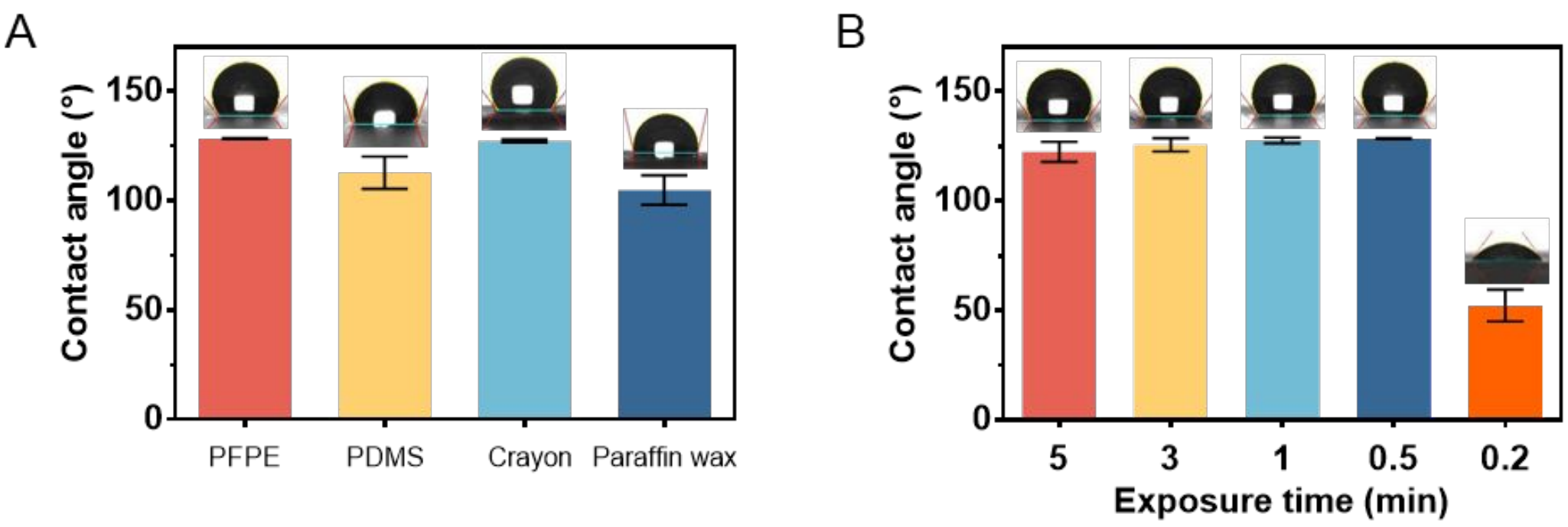

Figure S7. Hydrophobicity test using contact angle. (A) Comparing different materials. (B) Different UV treatment time for MD700 curing. Error bars represent standard deviation (n = 3).

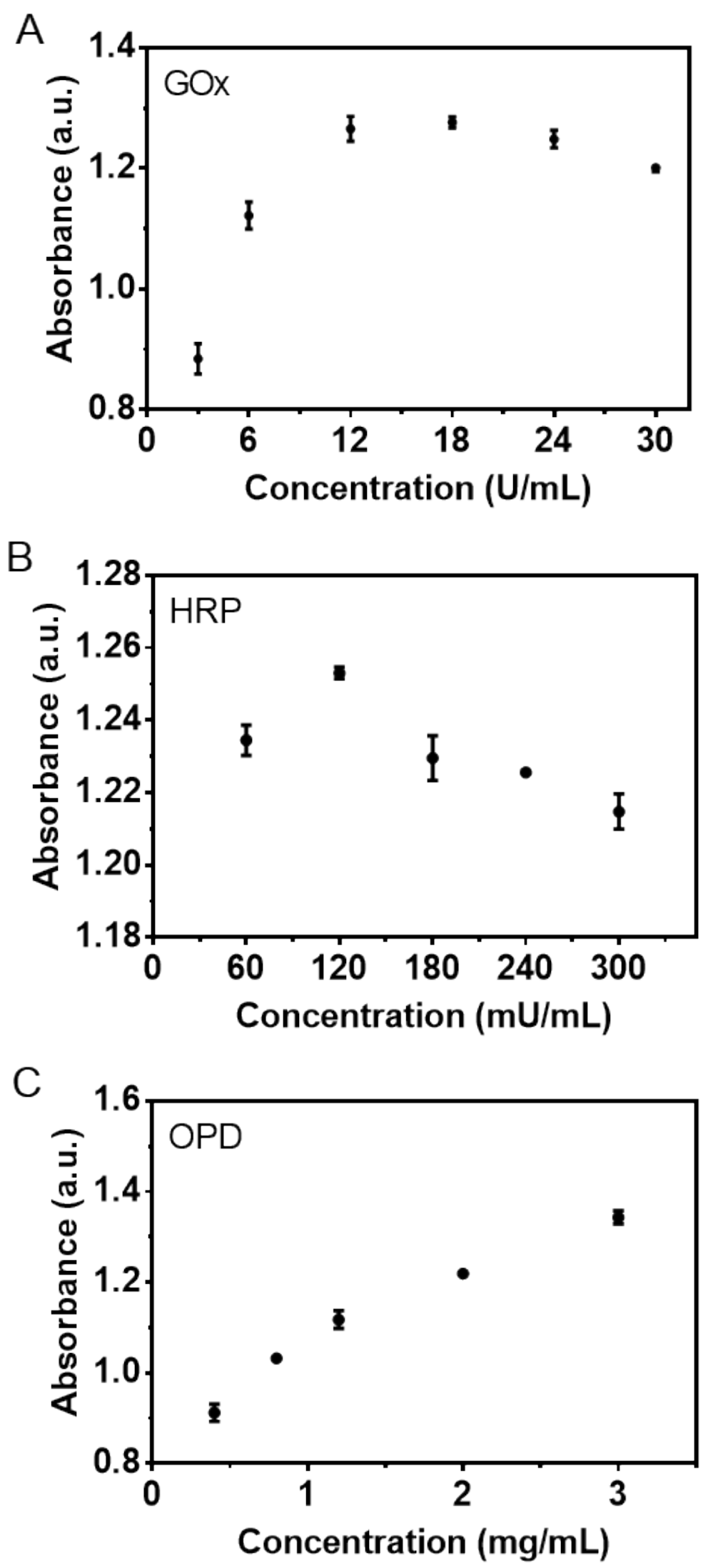

Figure S8. Optimization of enzymatic reagent concentrations for colorimetric glucose sensing. Optimization curves detailing the effect of (A) GOx concentration, (B) HRP concentration, and (C) OPD concentration on the spectrophotometric absorbance response at 420 nm. Error bars represent standard deviation (n = 3).

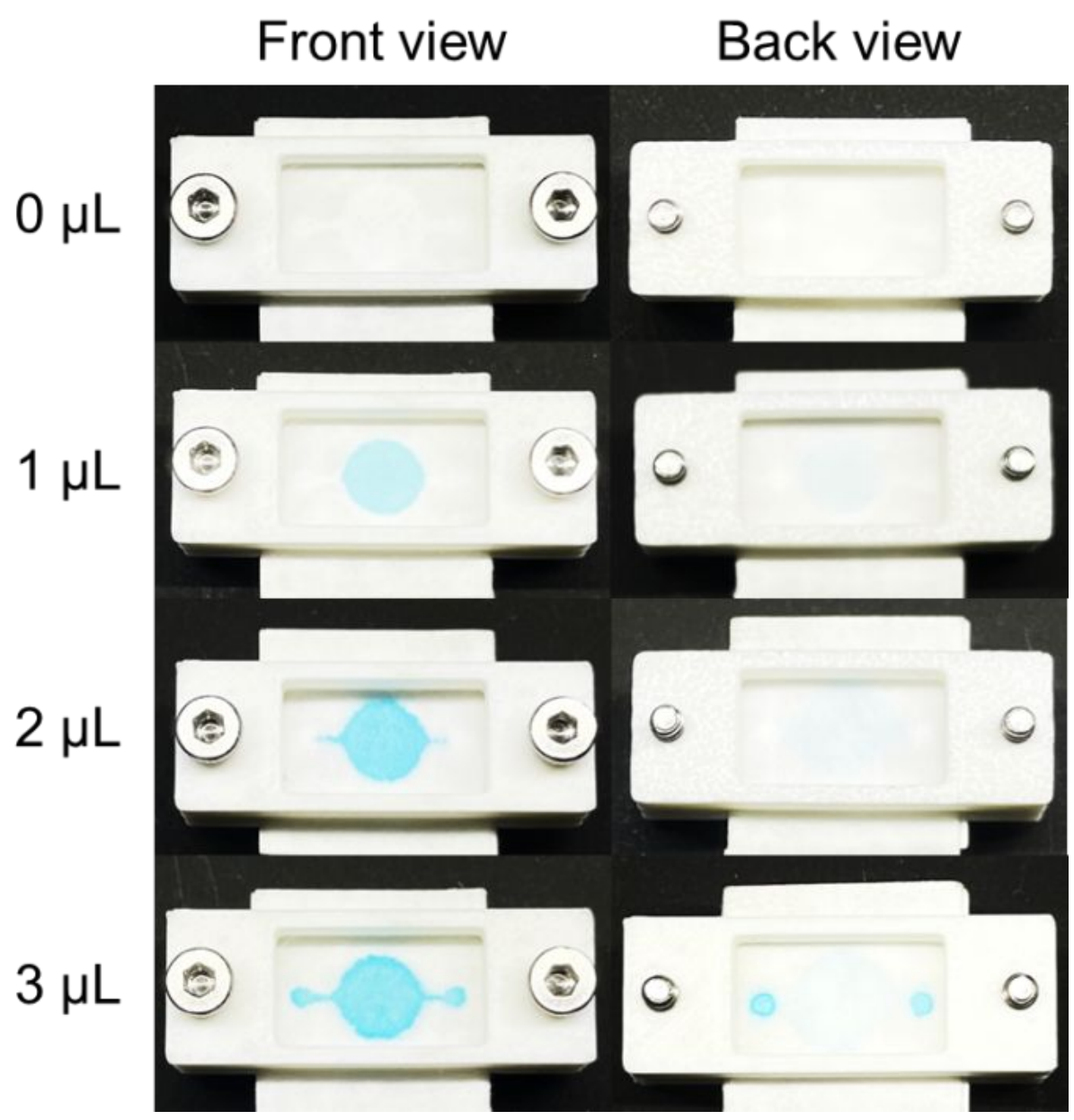

Figure S9. Channel coverage of DM-μPAD using blue dye at various sample volumes.

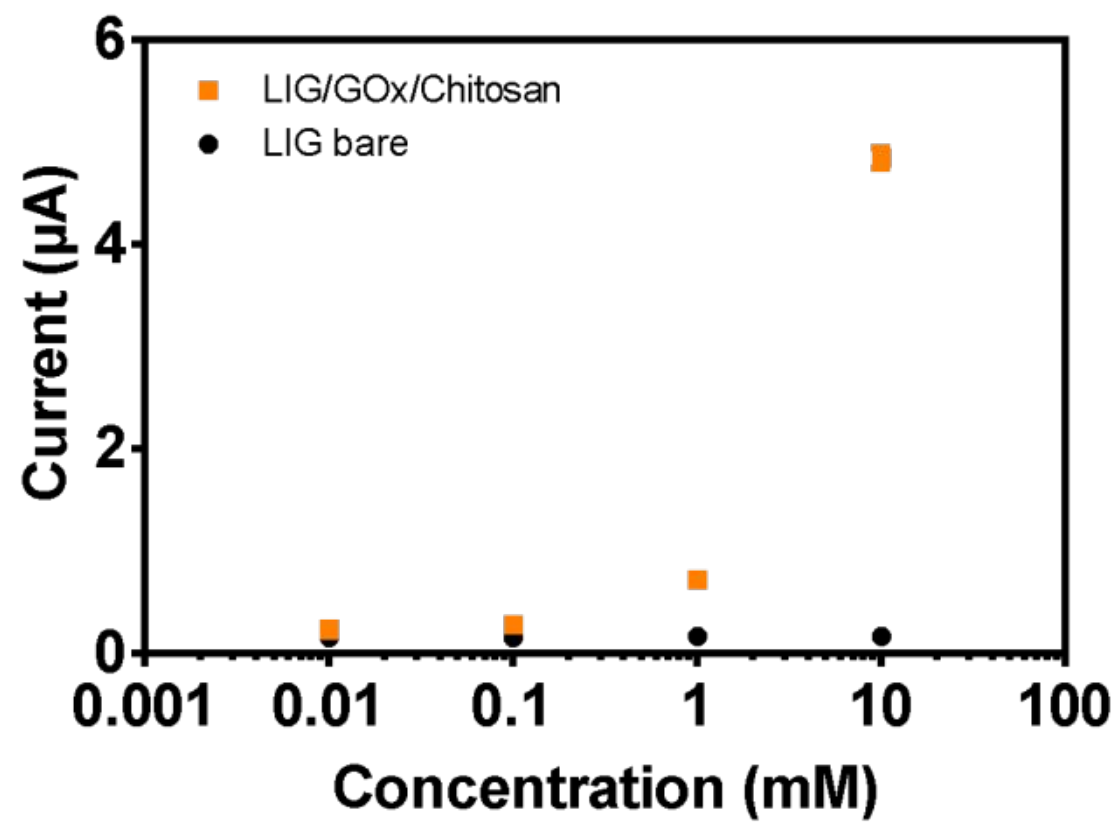

Figure S10. Current response with different concentrations of glucose between bare LIG and GOx/chitosan-modified LIG at applied potential 0.6 V.

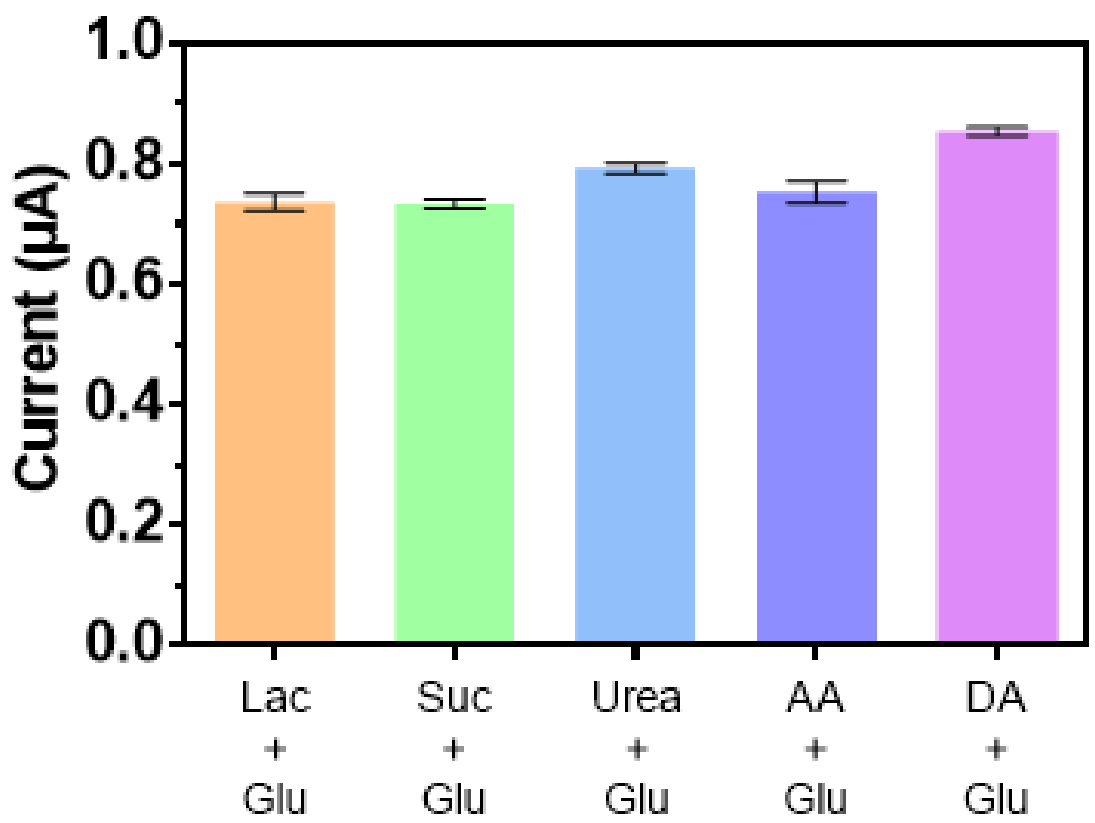

Figure S11. Evaluate glucose level in the presence of sweat interferents. Error bars represent standard deviation (n = 3).

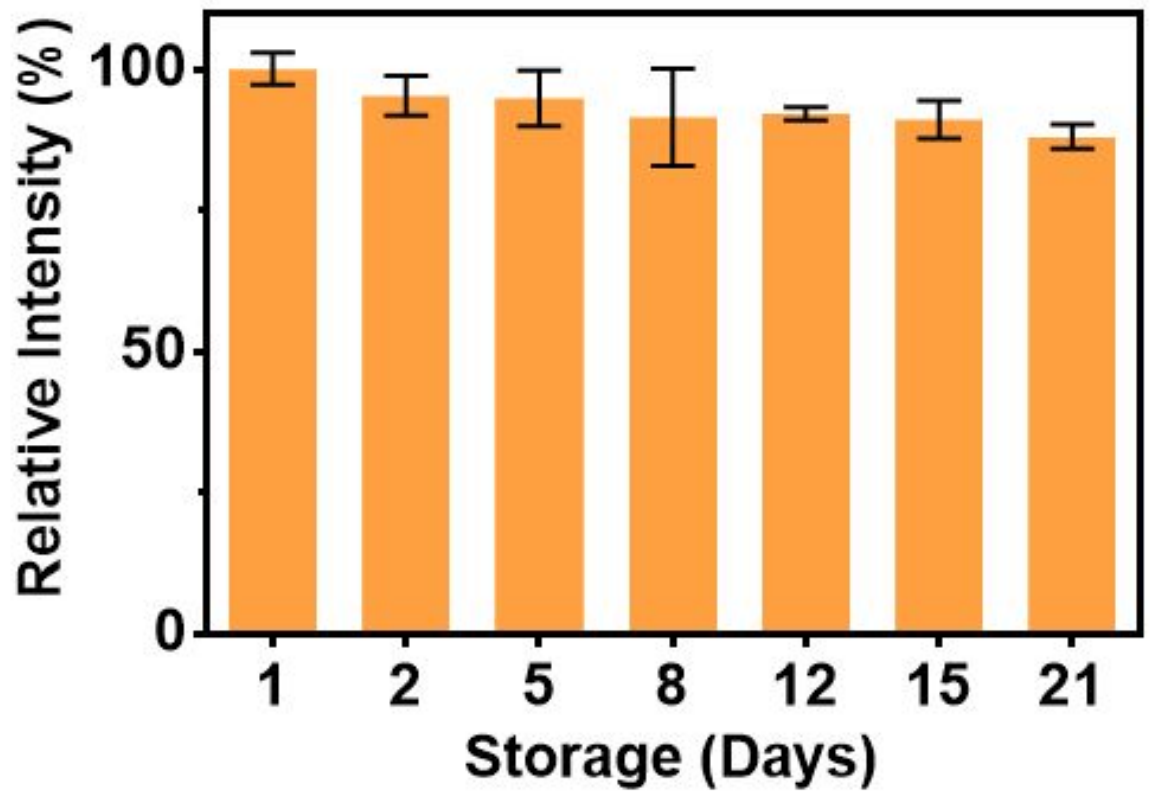

Figure S12. Relative current intensity (%) of the modified LIG working electrode toward glucose detection over 21 days. Error bars represent standard deviation (n = 3).

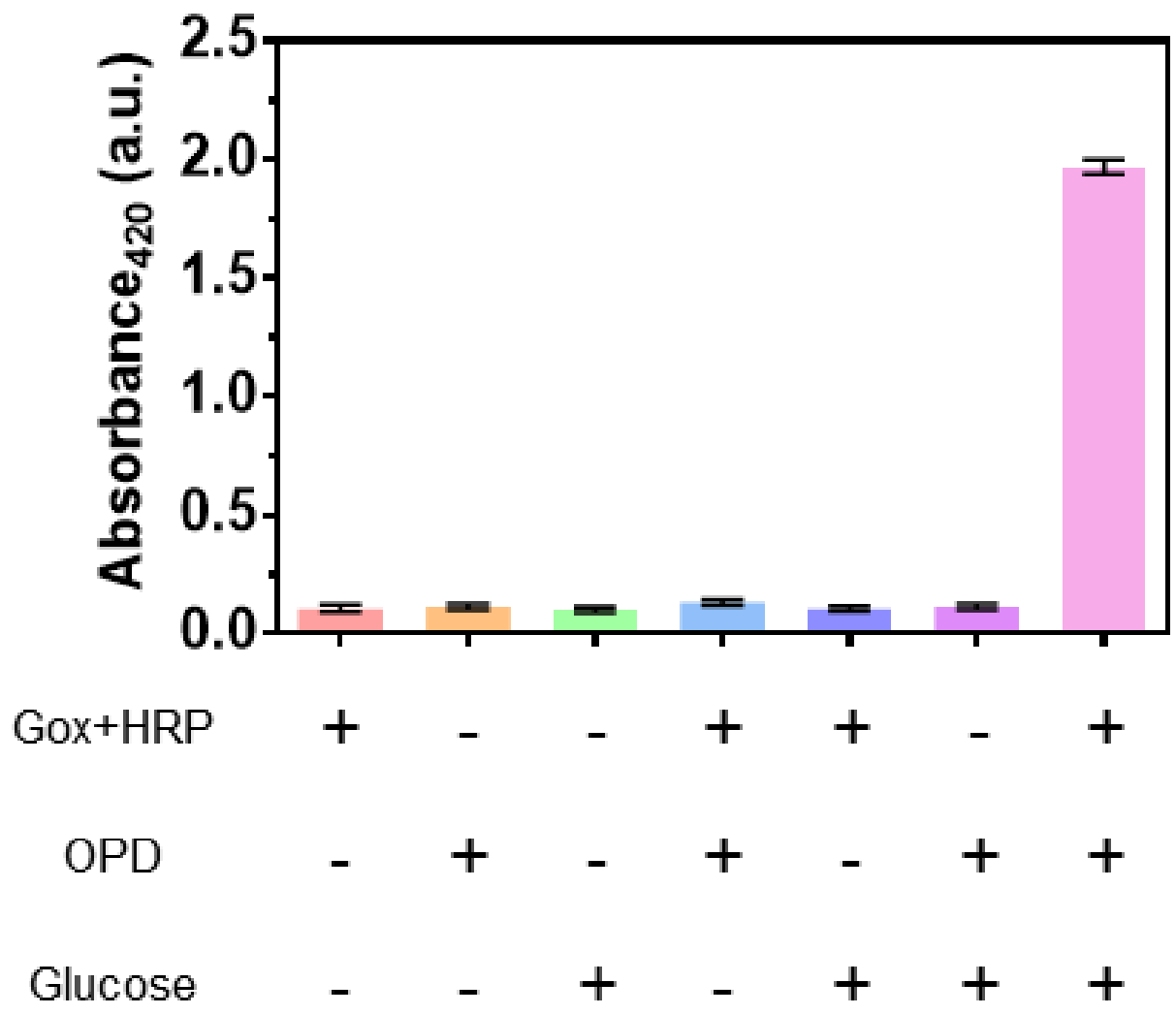

Figure S13. Feasibility of glucose detection system using colorimetry. Error bars represent standard deviation (n = 3).

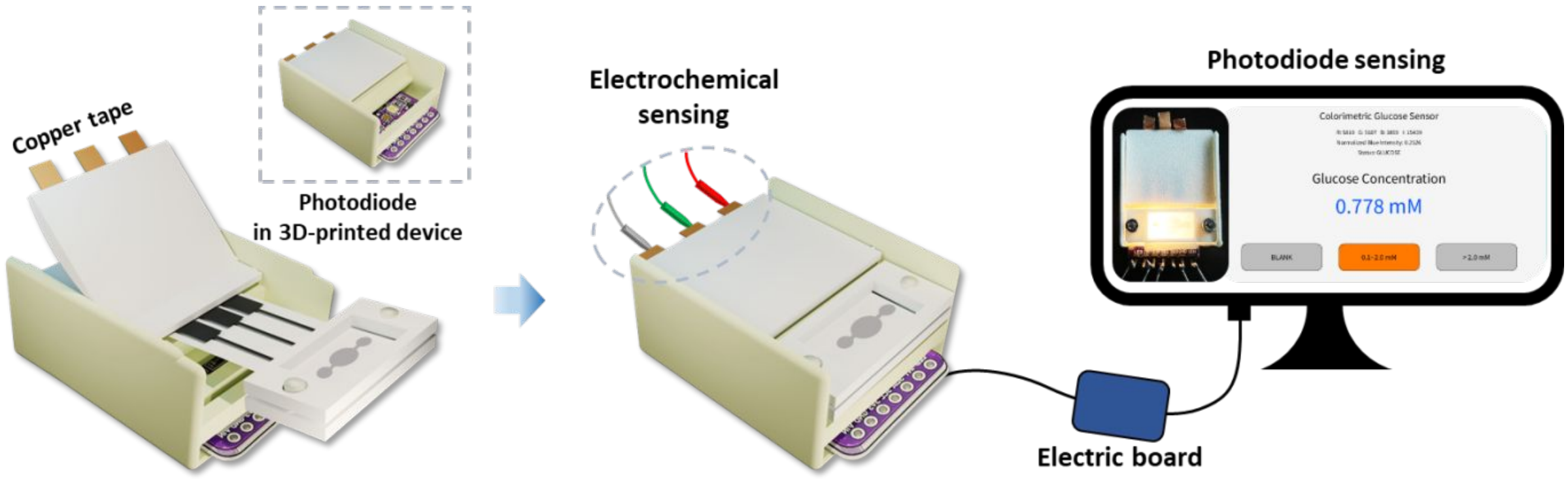

Figure S14. Schematic diagram of DM-μPAD in custom 3D printed device with photodiode.

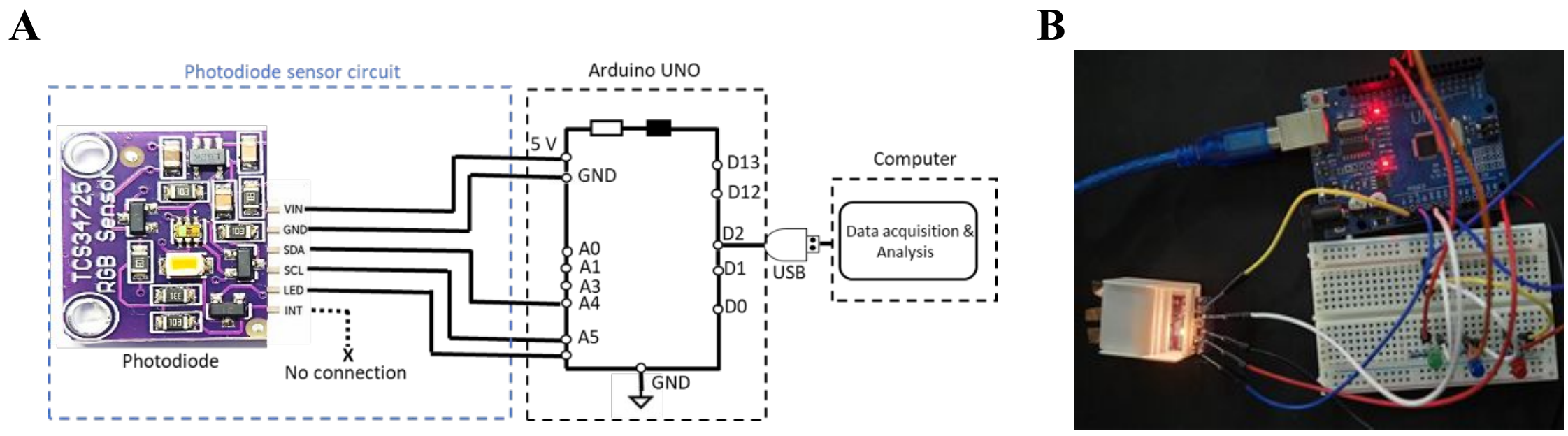

Figure S15. Portable photodiode-based detection system for colorimetric analysis. (A) Schematic illustration of the photodiode sensor circuit interfaced with an Arduino Uno microcontroller. (B) Photograph of the assembled portable detection system showing the Arduino Uno, breadboard circuitry, and 3D-printed sensing chamber used for colorimetric signal acquisition from the DM-μPAD.

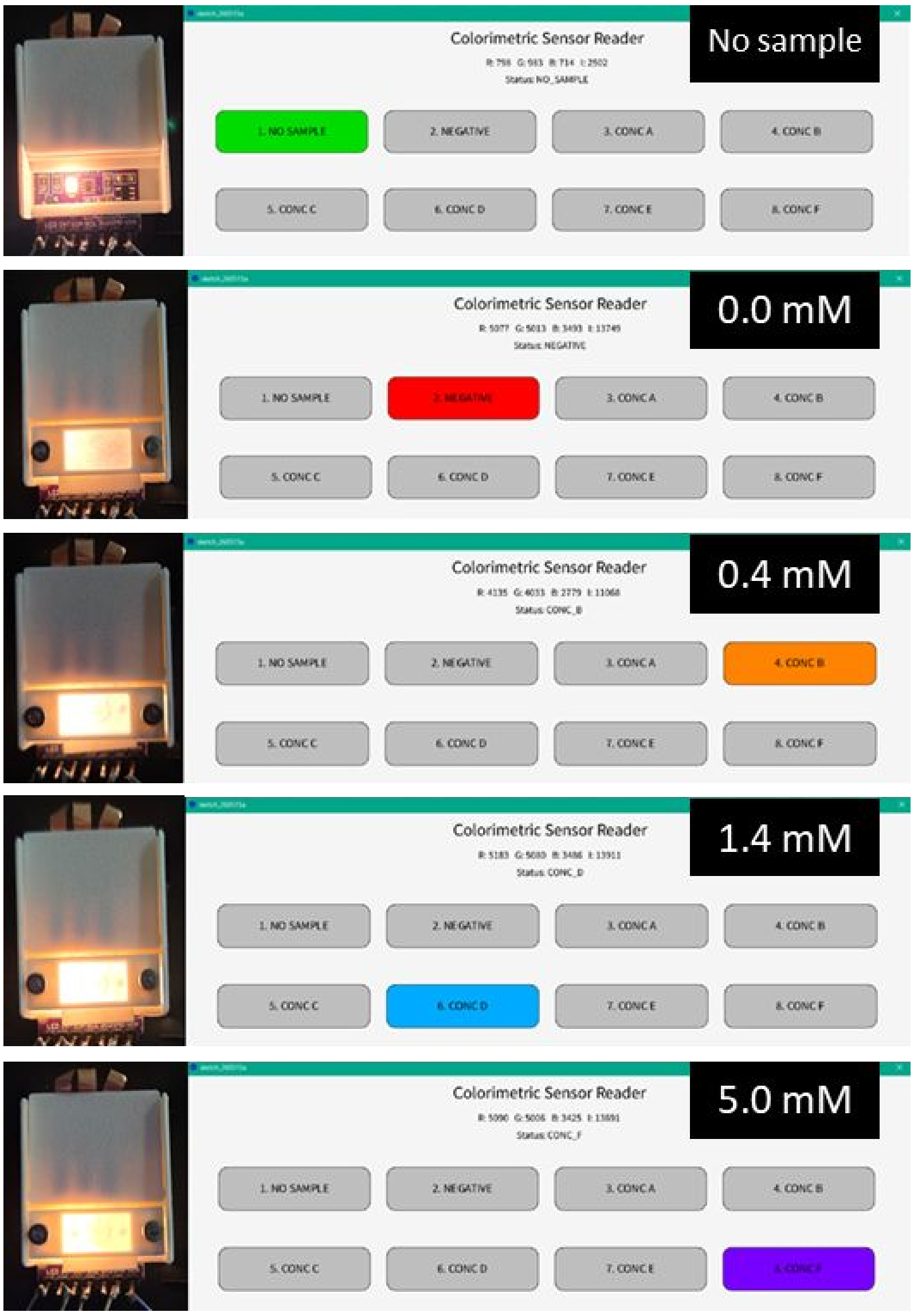

Figure S16. Portable photodiode-based colorimetric detection of glucose using the DM-µPAD. Representative screenshots of the Arduino-TCS34725 sensor interface displaying the classification of different colorimetric responses (Concentration order from A to F: 0.1, 0.4, 1.0, 1.4, 2.5 and 5.0 mM).

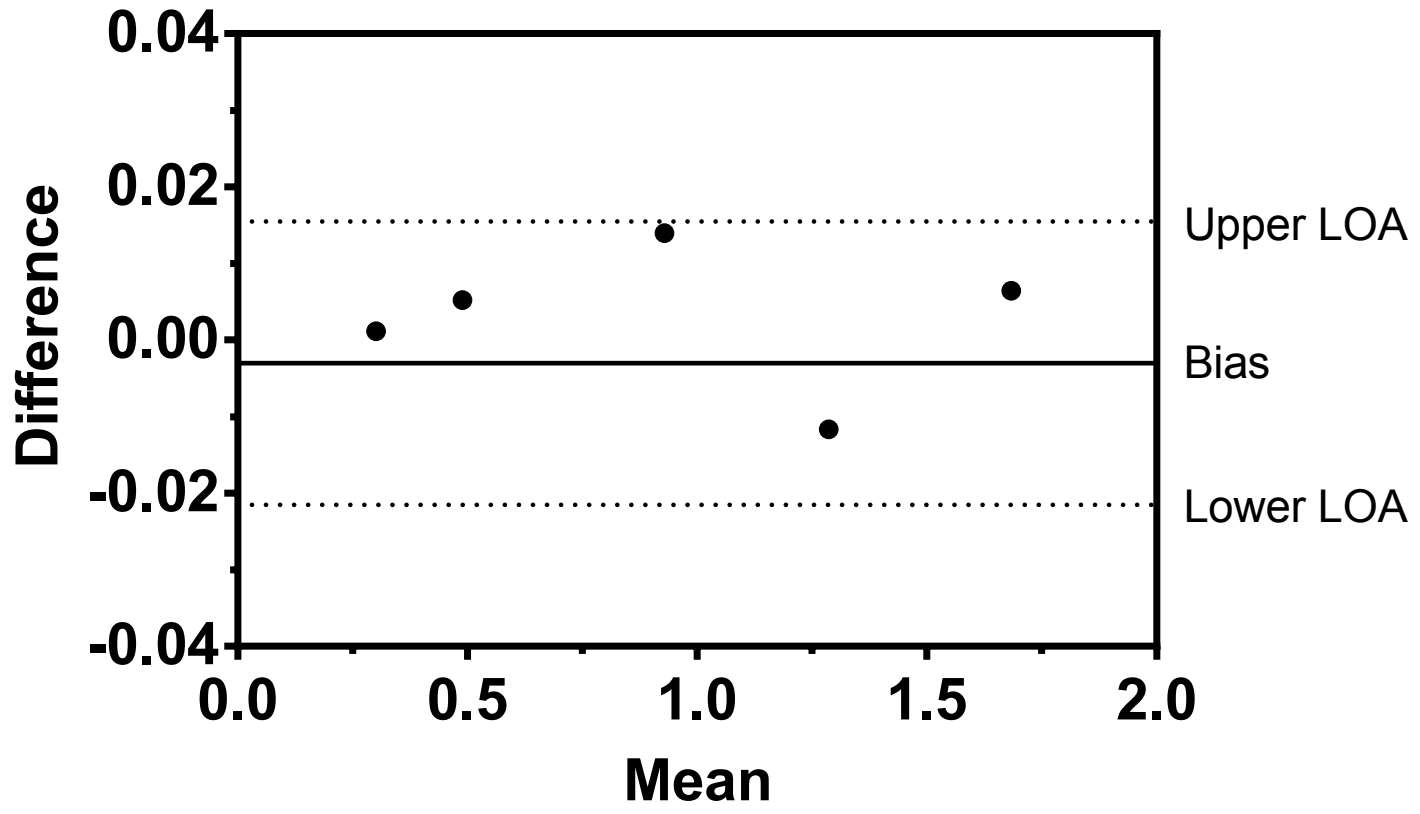

Figure S17. Bland–Altman analysis comparing glucose concentrations obtained from electrochemical and colorimetric measurements. The solid line represents the mean bias, while the dashed lines indicate the 95% limits of agreement (bias ± 1.96 SD).

Table S1. Comparison of electrochemical and colorimetric glucose sensor

Method	Analysis	Platform	Substrate	Linear range	LOD	Ref
Electrochemistry	Chrono -amperometry	PEDOT:PSS/Ti_3C_2/MWCN	Paper based graphite	0.01 -0.4 mM	7 μM	1
Electrochemistry	Chrono -amperometry	PB/GOx	Chitosan based LIG	25–300 μM	9.6 μM	2
Electrochemistry	Amperometry	Pt-NPs/Zein/Gel-GOx	Paper based LIG	0 - 2 mM	10 μM	3
Colorimetry	UV–visible spectra	GOx	PC2Ag nanofiber	0.1-1 mM	78.3 μM	4
Colorimetry	RGB	GOx@CuBDC, TMB	Paper	0.04–0.9 mM	20.7 μM	5
Colorimetry	Gray intensity	TMB, GOx, FN-GQDs	Paper	0.025-0.5 mM	18.75 μM	6
Electrochemistry	Chrono -amperometry	GOx, CS, PB	Paper based carbon paste	0 - 0.2 mM	126 μM	7
Colorimetry	Hue	PANi/ITO				
Electrochemistry	DPV	GOx, HRP, ABTS	SPE	0 – 0.5 mM	0.467 μM	8
Colorimetry	Euclidean distance		Paper	0.0625 – 0.5 mM	10 μM	
Electrochemistry	Chrono -amperometry	GOx/CS	Paper based LIG	0.01 - 3.0 mM	9.23 μM	This work
Colorimetry	RGB	OPD/HRP/GOx/CS	Paper	0.1 - 2.0 mM	57.9 μM	

PEDOT:PSS: Poly(3,4-ethylenedioxythiophene):poly(styrenesulfonate), **MWCN**: Multiwall carbon nanotube, **PB**: prussian blue, **PC2Ag nanofiber**: PVA-chitosan AgNPs nanofiber, **CuBDC**: copper 1, 4-benzenedicarboxylate, **TMB**: 3,3',5,5'-Tetramethylbenzidine, **FN-GQDs**: Ferric hydroxide/nitrogen-doped graphene quantum dots, **CS**: Chitosan, **PANi**: polyaniline, **ITO**: indium tin oxides, **ABTS**: 2,2'-azino-bis(3-ethylbenzothiazoline-6-sulfonic acid), **SPE**: Screen-printed electrode.

Reference

(1) Moradi, S.; Hosseini, M.; Firoozbakhtian, A.; Barshan Tashnizi, M.; Reza Ganjali, M. Electrochemical detection of glucose in sweat using a paper-based origami biosensor based on PEDOT:PSS/Ti_3C_2/MWCNT composite. *Journal of Electroanalytical Chemistry* **2024**, *973*. DOI: 10.1016/j.jelechem.2024.118675.
(2) Hamidi, H.; Levieux, J.; Larrigy, C.; Russo, A.; Vaughan, E.; Murray, R.; Quinn, A. J.; Iacopino, D. Laser induced graphene (LIG) biosensors derived from chitosan: Towards sustainable and green electronics. *Biosensors and Bioelectronics: X* **2023**, *15*. DOI: 10.1016/j.biosx.2023.100403.
(3) Sakdaphetsiri, K.; Thaweeskulchai, T.; Sukmas, W.; Wang, J.; Schulte, A.; Rodthongkum, N. Laser-induced graphene electrode modified by platinum nanoparticle/zein/gelatin/glucose oxidase for non-invasive glucose sensor in multiple biofluids. *Anal Chim Acta* **2025**, *1353*, 343974. DOI: 10.1016/j.aca.2025.343974.
(4) Mohanasundaran, A.; Kim, J. Colorimetric point-of-care glucose detection in sweat using paper-based strips with modified PVA-chitosan nanofibers. *Journal of Industrial and Engineering Chemistry* **2025**, *148*, 778-790. DOI: 10.1016/j.jiec.2025.01.037.
(5) Fan, B.; Wu, Y.; Guo, H.; Yu, F.; Liu, L. E.; Yu, S.; Wang, J.; Wang, Y. Self-assembly of cascade nanoenzyme glucose oxidase encapsulated in copper benzenedicarboxylate for wearable sweat-glucose colorimetric sensors with smartphone readout. *Anal Chim Acta* **2024**, *1316*, 342852. DOI: 10.1016/j.aca.2024.342852.
(6) Tran, H. V.; Nguyen, T. V.; Tran, L. T. A colorimetric paper-based analytical device for glucose detection using a ferric-based peroxidase nanozyme. *New Journal of Chemistry* **2026**, *50* (5), 2192-2198. DOI: 10.1039/d5nj04134a.
(7) Yeon, S. Y.; Seo, M.; Kim, Y.; Hong, H.; Chung, T. D. Paper-based electrochromic glucose sensor with polyaniline on indium tin oxide nanoparticle layer as the optical readout. *Biosens Bioelectron* **2022**, *203*, 114002. DOI: 10.1016/j.bios.2022.114002.
(8) Xu, J.; Shen, J.; Zhang, B.; Zhang, Y.; Lv, X.; Zhu, G. Smartphone-based portable electrochemical-colorimetric dual-mode biosensor for glucose detection in a co-reaction system. *Electrochimica Acta* **2024**, *481*. DOI: 10.1016/j.electacta.2024.143952.
